# The Role of Ascorbic Acid in the Process of Azo Dye Degradation in Aqueous Solution

**DOI:** 10.3390/molecules29153659

**Published:** 2024-08-02

**Authors:** Adrianna Pach, Aleksandra Zaryczny, Agnieszka Podborska, Magdalena Luty-Błocho

**Affiliations:** 1 AGH University of Krakow, Faculty of Non-Ferrous Metals, al. A. Mickiewicza 30, 30-059 Krakow, Poland; apach@agh.edu.pl (A.P.);; 2 AGH University of Krakow, Academic Centre for Materials and Nanotechnology, al. A. Mickiewicza 30, 30-059 Krakow, Poland; podborsk@agh.edu.pl

**Keywords:** azo compound, ascorbic acid, pollutants, methyl orange, tropaeolin OO, calcon

## Abstract

In this work, the role of ascorbic acid in the process of azo dye degradation was explained. For this purpose, the kinetics of azo dye degradation under different conditions was studied. Among them, the influence of daylight protection/exposition, different concentrations of ascorbic acid (0.567–0.014 mol/dm^3^), and temperature (20 °C and 50 °C) on the rate of the dyes’ degradation was considered. For this process, the kinetic equation was proposed, which indicates that the process of azo dye degradation using ascorbic acid is first order. Moreover, the observed rate constants were determined, and the mechanism of azo dye degradation was proposed. Spectrophotometry results, together with FTIR, fluorescence spectroscopy, and DFT calculations, explain the origin of the decolorization of the azo dyes and highlight the role of ascorbic acid in this process. Detailed analysis of the obtained products indicates that the process itself goes through several stages in which equally or more toxic compounds are formed. Obtained results from LCMS studies indicate that during tropaeolin OO degradation, 1,2-Diphenylhydrazine (*m*/*z* 185.1073) is formed. Thus, the process of azo dye degradation should be carried out in protective conditions. The proposed mechanism suggests that ascorbic acid at high content levels can be used for azo dye degradation from aqueous solution and can be an alternative method for their removal/neutralization from waste solution but with caution during the process.

## 1. Introduction

**Figure 1:**
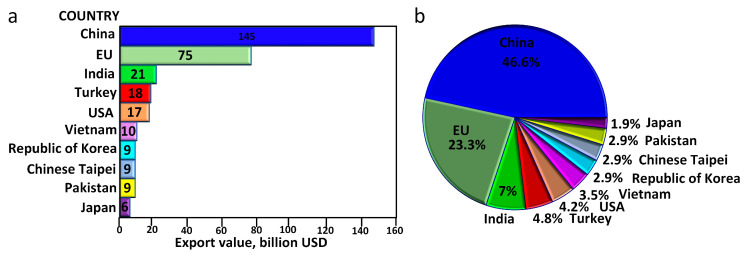
Top textile exporting countries worldwide 2021 (**a**) and share of the country in textile exports (**b**), adapted from [7].

Currently, azo dyes represent more than 60% of total global dye production. Due to their relatively low price, high availability, possibility of obtaining a wide range of colors, and high dyeing power, they represent the largest group of dyes used in industry applications [1,2,3]. The industry sectors using azo dyes are mainly the leather, printing, cosmetics, and food (coloring of sweets, jams, jellies, and ice creams) industries. However, the largest consumer of azo dyes is the textile industry [1,4]. The worldwide production of the textile industry reached USD 322 billion in the last year. Among countries that comprise the top textile exporters are China and India (see Figure 1), accounting for over 52% of global exports. The textile industry uses pigments, among which 65–75% are based on azo dyes [5]. Azo dyes are aromatic compounds containing in their structure nitrogen atoms connected by a double bond (AR-N=N-AR) [6].

The problems generated by the textile postproduction industry’s waste streams are dangerous for human health and the environment. Estimated in wastewater from the textile industry, the concentration of dyes varies from 2% to 50% [8]. Toxic dyes contained in wastewater cause significant pollution of water bodies hindering the transmission of sunlight into water. Therefore, oxygen saturation decreases, which disturbs the photosynthetic activity of aquatic plants [9,10]. Moreover, the dyes are toxic, carcinogenic, and mutagenic for living organisms [11]. Therefore, it is necessary to develop an effective azo dye removal and/or degradation method. Mostly, for this purpose, the physical, chemical, and biological approaches are applied [12]. The physical treatment includes adsorption, ion exchange, membrane filtration, coagulation, and flocculation techniques. Chemical treatments contain UV irradiation [13], electrokinetic coagulation [14] and filtration, Fenton reaction, ozonation [15], and the photochemical process [16]. These processes can be enhanced via catalyst application [17,18,19]. Whereas the biological approach includes biosorption and bioaccumulation [20].

Beyond the mentioned methods, a promising approach is the use of ascorbic acid as one of the components influencing azo dye degradation. Ascorbic acid has a wide range of other applications, including pharmaceutical, agricultural, food, and chemical reduction (organic and inorganic compounds), or in the process of nanoparticle formation [21,22,23]. Recent literature sources report that ascorbic acid is present as a component in systems analyzed for the degradation of azo dyes. Verma et al. [11] described the process of Reactive Black 5 removal involving the cobalt (II)/ascorbic acid/H_2_O_2_ system which achieved 97% degradation of azo dye within 15 min. The authors indicated that the fast removal of the dyes was possible due to the hydroxyl radicals formed during the reaction. The rate of radical production was dependent on the concentration of ascorbic acid and H_2_O_2_, but a mechanistic explanation was not included in the paper. Ou et al. [24] used ascorbic acid as a surface modifier for TiO_2_ semiconductors to decolorize methyl orange. The formation of ascorbic acid-based complexes on the semiconductor surface increased the rate of photocatalytic decolorization of methyl orange (both under sunlight and UV light irradiation). The modification of the surface shifts the absorption threshold of the semiconductor causing a more efficient solar spectrum in cleavage of the azo bond. According to the authors, the formation complexes are sites where charge transfer accumulates. Free electrons react with oxygen to generate peroxides, intensifying the oxidation of the azo dye. The color of methyl orange was removed with an irradiation time of 3 h (1 g/L of ascorbic acid and TiO_2_). Ascorbic acid was also used as a reducing agent for the synthesis of Au/AC catalysts. The presence of ascorbic acid was one of the factors influencing high catalytic activity. Gold nanoparticles supported on AC were used for catalytic reduction of methyl orange, Congo red, and Erichrome black T. The removal of azo dyes was due to catalytic degradation which occurred within several seconds. However, the obtained intermediate products (as indicated by the appearance of new absorbance peaks) were not identified [25]. The combination of ascorbic acid with Fe@Fe_2_O_3_ was used to intensify the Fenton reaction for the removal of organic impurities (including azo dyes) [26]. Singh et al. used ascorbic acid as an ingredient in the synthesis of chitosan-grafted-poly(acrylamide) adsorbent to remove dyes, i.e., Remazol violet. However, ascorbic acid was not found to be actively involved in the process of dye degradation [27]. Wakimato et al. [28] showed that irradiation with visible light (>420 nm) of a solution containing methyl orange in the presence of C60 fullerene adsorbed on silica gel and ascorbic acid leads to dye decomposition with 96% efficiency obtained within 25 min. It was also indicated that, during this process, N,N-dimethyl-p-phenylenediamine and a sulfanilic acid were generated.

In this context, the effect of solely ascorbic acid addition on the process of azo dye removal has not yet been described. Thus, in the present study, the effect of ascorbic acid on the degradation of azo dyes, namely methyl orange (MO), tropaeolin OO (TR), and calcon (CL), in aqueous solutions was tested. The effects of solar radiation, different concentrations of ascorbic acid, and temperature on the rate of dye degradation were presented. Moreover, the detailed mechanism of this process was described. The presented studies suggest that ascorbic acid in selected conditions can be applied for the removal of azo compounds from waste solution while maintaining caution regarding hazardous intermediates.

## 2. Results and Discussion 

### 2.1. Experimental Conditions 

The process of azo dye removal was carried out with different amounts of ascorbic acid (0.01 g–0.4 g) and temperatures (20 and 50 °C), see Table 1. The influence of daylight exposition (DLE) on the rate of dye degradation was also studied. In the experiments, the appropriate amount of ascorbic acid was added to glass vials containing 4 mL of azo dye solutions (MO, TR, and CL) with a concentration of 5 × 10^−5^ mol/dm^3^. Then, mixed reactants were shaken (about 1 min) until the ascorbic acid was completely dissolved. The amount of ascorbic acid was selected, taking into account the solubility of this compound in water, i.e., 300 g/L at 20 °C [29]. Thus, the maximal proportion of ascorbic acid to solution volume was set below this value, i.e., a max of 0.1 g/mL. To check the temperature influence on the process of azo dye degradation, the selected samples were kept in the thermostatic bath at 50 °C. To verify the daylight sensitivity of azo dye solutions containing ascorbic acid, several samples were covered with protective material and placed in a dark place (DLP—daylight protection). Otherwise, the sample was exposed to the sunlight. This exposition was limited to light naturally entering the laboratory through the window (these experiments were carried out in July when the highest sunshine occurs at about 221.5 h, while the total solar irradiance in Krakow is 596.7 MJ·m^−2^ [30]. In the predetermined time intervals, all solutions were analyzed using spectrophotometry UV-Vis, whereas the change in the solution color was documented using a Nex Sony camera (Sony, Tokyo, Japan). All experimental conditions have been gathered in Table 1.

### 2.2. Spectra of Reagents and Molar Coefficients Determination

**Figure 2:**
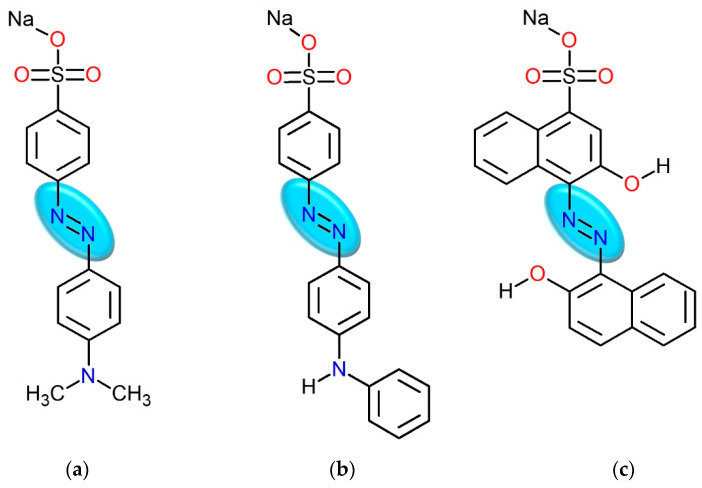
The chemical structures of the azo dyes used in the experiments with a marked azo group: methyl orange (**a**); tropaeolin OO (**b**); and calcon (**c**).

Before the commencement of the process of azo dye degradation, all reagents were analyzed using spectrophotometry. The registered UV-Vis spectra for aqueous solutions of methyl orange, tropaeolin OO, calcon (Figure 2), and ascorbic acid (H_2_O as solvent) allowed for the determination of the wavelength range at which processes can be monitored without the risk of overlapping of spectra from substrates and/or formed products.

In the case of ascorbic acid, the spectrum with one strong maximum localized at wavelength 246 nm was registered (see Figure S1a, Supplementary Materials). This peak is close to that at 243 nm (ε_243nm_ = 9650 M^−1^cm^−1^, at pH = 1) provided in the literature, and it is assigned to H_2_Asc (an undissociated form of ascorbic acid) [31]. However, in our condition, with a pH of about 3 (water as solvent), the determined value of the molar coefficient equals 1732 M^−1^cm^−1^ (see Figure S1b, Supplementary Materials). The value of the wavelength is also in good agreement with the spectrum for this form calculated with TD-DFT methods for different forms of ascorbic acid (see, Figure S1c). Whereas the characteristic spectra for azo dyes have more than one characteristic peak (maximum value of absorbance at selected wavelength). The tropaeolin OO in the studied concentration range has two maxima localized at 272 and 445 nm under the studied pH range between 4 and 5 [32] (see Figure S2a, Supplementary Materials), for which the values of the molar coefficient were determined (see Figure S2b, Supplementary Materials). Through comparison of these experimental spectra with TD-DFT calculated spectra (with maxima located at 274, 331, and 480 nm, see Table S1, Supplementary Materials) we can describe the first maxima as a HOMO to LUMO+2 transition and the second one as a HOMO–LUMO transition (see Figure S2c, Supplementary Materials). The HOMO orbital is localized mainly on two benzene rings connected by a nitrogen atom, whereas the LUMO is localized on a benzene ring with a sulfonic group (Figure S2d, Supplementary Materials).

The methyl orange, depending on the pH, might have two peaks located at 275 and 470 nm at pH = 4.1 and three peaks located at 277, 319, and 504 nm in acidic conditions (pH 2.8). A detailed study of these molecules is described in our previous paper [33]. UV-Vis spectra were also registered for calcon and are characterized by three maxima of varying intensity (see Figure S3a, Supplementary Materials). Two strong peaks are located at the wavelengths 218 nm and 544 nm, and one is less intense at 340 nm. According to the theoretical spectrum calculated for the calcon molecule, the peak at 544 nm is connected with the HOMO–LUMO transition (see, Figure S3c,d, Table S2, Supplementary Materials).

From the obtained UV-Vis spectra for all dyes (Figures S1a, S2a and S3a, Supplementary Materials), the graphs showing the dependence of absorbance as a function of concentration were drawn (Figures S1b–S3b, Supplementary Materials). From the slope of the linear fit to the experimental data, the values of the molar coefficient were determined and summarized in Table 2.

Based on the obtained results, it can be concluded that, in the studied concentration range, spectra at shorter wavelengths coming from ascorbic acid and azo dyes overlap. Thus, the reaction taking place between reagents might be observed only at longer wavelengths, i.e., at 445 nm for TR, 465 nm for MO, and 544 nm for CL. In all cases, these peaks are connected with the HOMO–LUMO electron transition.

### 2.3. The Process of Azo Dye Degradation 

The possibility of the azo dyes’ degradation was tested using three selected compounds, tropaeolin OO, methyl orange, and calcon. The structures of these azo compounds are shown in Figure 2 and characterized spectrophotometrically in Section 2.1. Obtained results show that, at similar pH conditions, these compounds have a different optical response and, among them, calcon has the most extensive structure (Figure 2). These structural differences might influence the rate of dye degradation using ascorbic acid and also provide insight into the mechanism of this process. 

**Figure 3:**
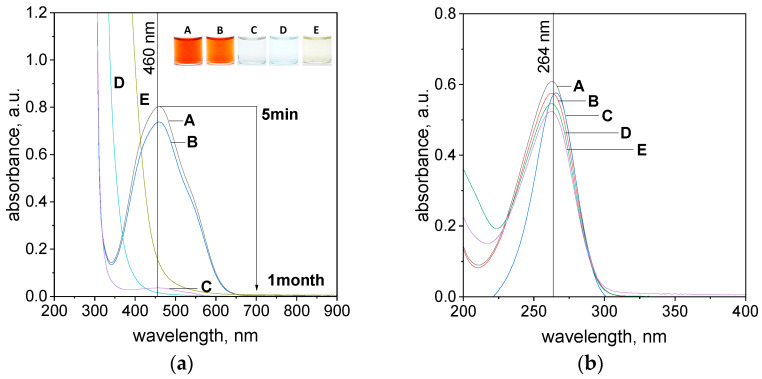
The UV-Vis spectra of the solution containing the mixture of 4 mL of tropaeolin OO (TR) mixed with 0.4 g of ascorbic acid (**a**); the spectrum of ascorbic acid after the ten-thousandth dilution (**b**) registered over time, A—5 min, B—1 h, C—24 h, D—48 h, and E—1 month. Conditions: C_0, MO_ = 5 × 10^−5^ mol/dm^3^ (the value of concentration before mixing), T = 20 °C, and path length of 1 cm.

The study of the process of azo compound degradation in aqueous solutions was started with a significant excess of ascorbic acid (0.4 g), and the process was carried out at 20 °C. After reagent mixing, i.e., the solution containing dye and the ascorbic acid, the change in color coming from TR, MO, and CL was observed (see Figure 3, Figure S4a,b, Supplementary Materials). For these samples, the UV-Vis spectra were registered. In all cases, a red shift of the maximum was observed. In the case of TR, it was moved from 445 to 460 nm and from 465 to 510 nm for MO. Whereas, for CL a blue shift (move of the peak position from 544 to 540 nm) was observed. These changes relate to the pH change of the solution after ascorbic acid addition. For example, in the case of TR, the pH of the solution changes from 5 to 2 after ascorbic acid addition (0.4 g into 4 mL of dye solution), see Supplementary Materials Table S3. It is known from the literature that the pH of the solution has a significant influence on the share of the azo dye forms present in the solution, which is described in detail elsewhere [34,35], and on ascorbic acid species. With time, the spectra evolution for TR (Figure 3) and other compounds (see Figure S4a,b, Supplementary Materials) were observed. It confirms that, between azo dyes and ascorbic acid, a chemical reaction takes place.

Detailed analysis shows that the rate of color change was dependent on the investigated azo dyes. In the case of tropaeolin OO, a rapid process of color degradation was observed. The intense orange color (A, B, Figure 3a) fades within 24 h (C, Figure 3a) and completely disappears after 48 h (D, Figure 3a). At this point, it is worth mentioning that spectrum characteristics for ascorbic acid also change with time. The concentration of this compound is so high that it was impossible to achieve spectrum registration (Figure 3a). Moreover, after 48 h and later on, an additional redshift (from the C to D and next to the E position, Figure 3a) of the spectrum was observed. For this purpose, the samples were additionally diluted ten thousand times to observe the spectrum response only from ascorbic acid. The characteristic maximum for this compound appears at 264 nm (A, Figure 3b) and it relates to its monodissociated form. This was confirmed also through TD-DFT calculations (Figure S1c). The intensity of this spectrum decreases, and it suggests that further reactions take place with time. Interestingly, there is also a redshift (C, Figure 3b), which might suggest the presence of additional oxidation of ascorbic acid compounds. The increase of the absorbance value at 200 nm suggests the presence of DHA in the solution, which has a maximum at 185 nm [36]. It reaches a maximum 48 h later (D, Figure 3b). Next, this value decreases, and it suggests further processes related to the reactivity of DHA. To confirm our supposition, we performed additional tests using FTIR spectroscopy. The obtained spectra evolution is shown in the Supplementary Materials (Figure S4c–f), and three main changes in the spectrum were observed within one week. First of all, the peak at 1066 cm^−1^ related to the vibration of the C-O bond in ascorbic acid disappeared. The second change involved the peak visible at 1500 cm^−1^ and assigned as the vibration of the O-H bond in monodissociated form which also disappeared. The last change was observed at 1753 cm^−1^. This peak was connected with C-O-H and switched to 1799 cm^−1^ (C=O bond vibration). All these changes suggest the formation of DHA and it is in good agreement with the result shown in [37]. The analysis of the solutions 1 month later showed that the sample turned yellow (E, Figure 3a), but no recovery of the spectrum from the dye was observed, which confirms that the process of dye degradation is irreversible. This suggests that the color of the solution is coming only from ascorbic acid and its oxidizing forms [36,38]. To confirm our supposition, we performed additional tests. During these studies, the behavior of ascorbic acid solution over time was observed. For this purpose, samples containing different concentrations of ascorbic acid (0.567 mol/dm^3^, 0.284 mol/dm^3^, and 0.0002 mol/dm^3^) were kept at a constant temperature, i.e., 50 °C for 1 week (see Figure S5a–c, Supplementary Materials). Then, solutions were analyzed spectrophotometrically at a certain period of time (5 min, 1 h, 24 h, and 7 days). It was observed that a fresh aqueous solution of ascorbic acid is colorless (see Supplementary Materials, Figure S5). Within a week, the samples containing 0.567 mol/dm^3^ and 0.284 mol/dm^3^ of ascorbic acid turned yellow (Supplementary Materials, Figure S5a,b). Whereas the samples containing 0.0002 mol/dm^3^ of ascorbic acid remained colorless (Supplementary Materials, Figure S5c). All samples were analyzed using spectrophotometry, and the obtained results are shown in the Supplementary Materials, Figure S5. Samples containing a high ascorbic acid content were additionally diluted ten thousand times to register a UV-Vis spectrum. The obtained results are shown in the Supplementary Materials, Figure S5a,b. Analysis of the solutions during aging but before dilution revealed red shifts in the spectrum. The registered spectrum after sample dilution has a characteristic maximum at 264 nm, whereas a characteristic peak at 257 nm was recorded for the 0.0002 mol/dm^3^ solution of ascorbic acid, whose intensity decreases with time. Moreover, the increase in absorbance value at 200 nm and isosbestic point appearance at 220 nm were also observed. After 7 days, a redshift and a new maximum at 293 nm were observed (Supplementary Materials, Figure S5c). Based on these observations, it can be concluded that the yellow color of the solution obtained after the week comes from the product of ascorbic acid oxidation.

**Figure 4:**
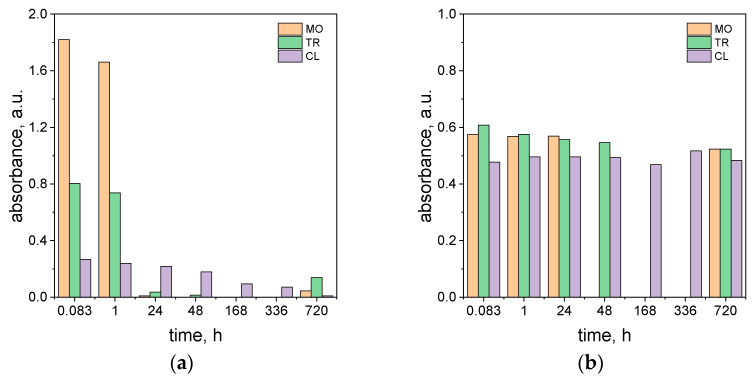
The change in the absorbance value coming from MO, TR, and CL and ascorbic acid at the λ characteristic for the respective dyes (510 nm for MO; 460 nm for TR, and 530 nm for CL) (**a**) and at 264 nm after 10,000 dilutions (**b**) at different times. Conditions: m_ascorbic acid_ = 0.4 g, C_0, MO, TR, CL_ = 5 × 10^−5^ mol/dm^3^ (the value of concentration before mixing), T = 20 °C, and path length of 1 cm. Please note that MO data were not collected after 48, 168, and 336 h or for TR after 168 and 336 h.

To compare the rate of the different azo dyes’ (TR, MO, and CL) degradation, the change in intensity of the absorbance at the λ_max_ characteristic for each of the dyes by time is shown in Figure 4a. Whereas the same dependency (at 264 nm) for ascorbic acid was shown in Figure 4b.

The comparison of the degradation of the different dyes throughout time (Figure 4a) at the highest value of ascorbic acid shows that the process rate is in the order MO > TR ≫ CL. 

The process of MO removal was faster than for TR, and the intense red color coming from methyl orange disappears after 24 h (see Figure 4a, Supplementary Materials, Figure S4a). The fading of the color was accompanied by a change in the UV-Vis spectrum as shown in Figure 4a and Supplementary Materials, Figure S4a. The characteristic maximum at 510 nm decreases over time (Supplementary Materials, Figure S4a). The process of MO and TR degradation occurs relatively faster than for the solution containing calcon. In the case of CL, the pink color coming from the dye disappears 1 month later (see Supplementary Materials, Figure S4b). For this dye, the registered spectrum has one maximum localized at a wavelength of about 530 nm, and its intensity decreases over time (see Figure 4a and Supplementary Materials, Figure S4b). The solutions containing MO and CL were also diluted to record the ascorbic acid spectrum. The appearance peak at 264 nm indicates the presence of a dissociated form of ascorbic acid in the solution. The MO samples were analyzed after 1 month. Similar to the previous observation (TR, Figure 3a), the samples appear pale yellow in color (see Supplementary Materials, Figure S4a). The small absorbance increases after a long time (i.e., 720 h), and it comes from oxidized products of ascorbic acid. The presence of these compounds in the solution starts to absorb light at 550 nm for the MO solution (see Figure S4a, Supplementary Materials). The graph shown in Figure 4b also suggests that the value of ascorbic acid slightly changes with time (see Figure 4b). However, we need to take into account a large dilution of the sample, in which small changes are a huge change in absorbance intensity.

### 2.4. The Influence of the Ascorbic Acid Concentration on the Process of Azo Dye Degradation

**Figure 5:**
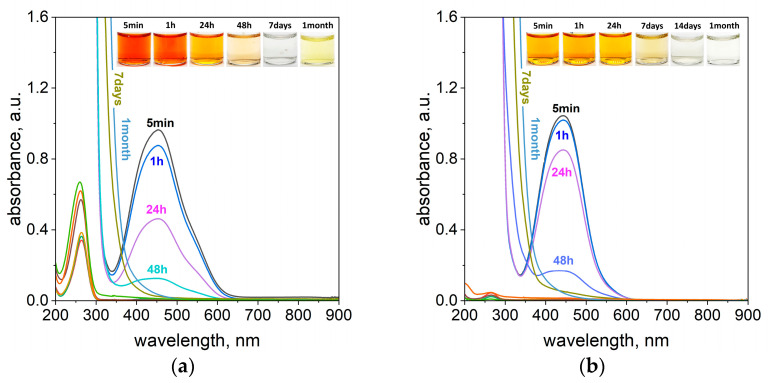

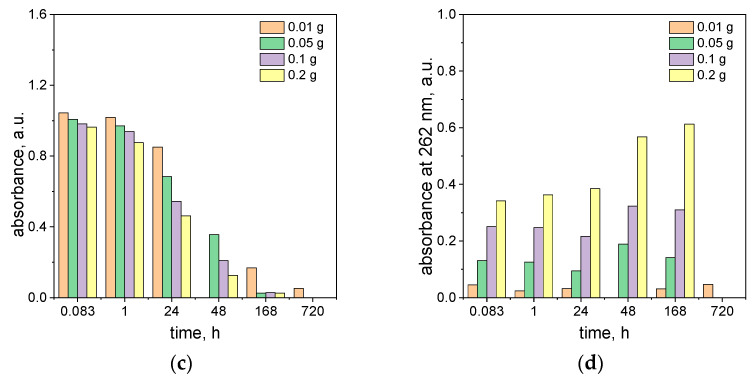
The UV-Vis spectra of the solution containing the mixture of 4 mL tropaeolin (TR) and ascorbic acid with different amounts of ascorbic acid: 0.2 g (**a**) and 0.01 g (**b**). The change in the absorbance value coming from TR (**c**) and ascorbic acid (after 10,000 dilutions) (**d**) with time at the different initial ascorbic acid amounts (0.01–0.2 g). Conditions: C_0,TR_ = 5 × 10^−5^ mol/dm^3^ (the value of concentration before mixing), T = 20 °C, and path length of 1 cm.

Taking into account the promising results obtained in Section 2.2. related to fast methyl orange and tropaeolin OO degradation, we wanted to determine the minimum value of ascorbic acid at which the process is still efficient. For this purpose, the process of MO and TR degradation was carried out at different ascorbic acid amounts, i.e., 0.2–0.01 g (see Table 1), and at a constant temperature (20 °C). After reagent mixing, the progressive fading of color coming from azo dyes was observed. According to a previous study, the spectra were registered after a certain period of time to register suitable changes. The registered changes in the color of the sample containing the highest and lowest content of ascorbic acid and registered spectra are shown in Figure 5a,b and in Supplementary Materials, Figures S6a,b and S7a–d, for other studied ascorbic acid amounts.

In the case of TR, the samples containing 0.05 g or more had an intense orange color (Figure 5a, Supplementary Materials, Figure S6a,b), while the sample with the lowest ascorbic acid content was intensely yellow (Figure 5b). The intensity of the color relates to the pH of the solution [32] which slightly changes depending on the amount of ascorbic acid. The share of the forms being in the azo dye equilibrium also changes, and it is visible on the “shape” of the spectrum. Comparing the highest and lowest ascorbic acid addition, from the spectra shown in Figure 5 it can be seen that the peak (Figure 5a) is not a Gaussian distribution. The share of the forms being in the equilibrium can be calculated after spectrum deconvolution, which was conducted in Origin 2021b software and is shown in Supplementary Materials, Figure S8. The peak deconvolution shows that, in fact, we have two peaks at 446 and 549 nm (see Supplementary Materials, Figure S8). Each of them relates to another form of TR [32]. With time, the color of the samples faded. Complete degradation of the azo dye for 0.2, 0.1, and 0.05 g of ascorbic acid occurred after 7 days (see Figure 5a and Supplementary Materials, Figure S6a,b). However, for the sample containing 0.01 g of ascorbic acid, the color turned bright yellow after 7 days and disappeared after 14 days (see, Figure 5b). The UV-Vis spectra registered for the solutions containing 0.2 g of ascorbic acid have a maximum located at 454 nm, see Figure 5a, while the sample for 0.1 g has a maximum at 450 nm (see Supplementary Materials, Figure S6a). For 0.05 g of ascorbic acid, the maximum is localized at 447 nm (see Supplementary Materials, Figure S6b). Whereas, at the lowest ascorbic acid content (0.01 g), the maximum peak was located at 443 nm (see, Figure 5b). The red shift of the peak was expected, taking into account that the pH in the solution slightly changes with the ascorbic acid concentration in the solution. The rate of decrease in absorbance value at a selected wavelength is shown in Figure 5c and its intensity was related to the amount of ascorbic acid in the solution. The disappearance of the characteristic peak occurs after 7 days for the sample with 0.2 g, 0.1 g, and 0.05 g of ascorbic acid (see Figure 5a and Supplementary Materials, Figure S6a,b), and for the sample with 0.01 g of ascorbic acid, the disappearance occurs after 14 days (see Figure 5b). Dilutions of the samples were made to register the characteristic peak coming from ascorbic acid. As in previous experiments, the peak was localized at 264 nm. This peak can be assigned as HAsc^−^, and it is in good agreement with the literature [31]. Similar experiments were performed for MO and are described in detail in the Supplementary Materials (Section S3, Figure S7).

To better overview the process of azo dye degradation, the rate constants were determined based on kinetic data and a fitted curve (see Supplementary Materials, Figure S9a,b). The character of the kinetic curve is exponential, and it suggests that the process of TR degradation is first order and can be described by the following kinetic equation with the rate constant *k*:

(1) $$\frac{dC_{dye}}{dt}=-k\cdot C_{AA}{\cdot C}_{dye}$$

Taking into account that in the solution we have a great excess of ascorbic acid (*AA*) compared to dye ($C_{AA}\gg C_{dye})$, the process of azo compound degradation is pseudo-first order, and the observed rate constant can be calculated from the following relation:

(2) $$k_{obs}=k\cdot C_{AA}\;$$

The solution of (1) has the following form:

(3) $$C_{dye}=C+\exp(-k_{obs}\cdot t)$$

where $C_{dye}$ is the concentration of dye at the time “*t*”, *C* is a constant, $k_{obs}$ is the observed first-order rate constant, k is the second-order rate constant, and *t* is the time.

The values of the determined rate constants are gathered in Table 3.

The second-order rate constant can be determined from the slope of the linear equation fitting to the experimental data, i.e., *k*_obs_ = f(*C_AA_*), see Supplementary Materials, Figure S9c. These values equal 0.06 and 0.32 Mh^−1^ for TR and MO, respectively.

### 2.5. The Influence of Temperature on the Process of Dye Removal

The Arrhenius equation shows the relation between kinetic rate constants with temperature according to Equation (4):

(4) $$k=Ae^{-\frac{E_{A}}{RT}}$$

where *k* is the rate constant (s^−1^); *A* is the pre-exponential factor; *E_A_* is the activation energy; *R* is the gas constant, 8.314 J/(mol·K); and *T* is the temperature (K).

According to the Arrhenius dependency, the process of azo dye degradation should be faster at higher temperatures. Taking into account that all our experiments were carried out at 20 °C, we also performed experiments at a higher temperature, i.e., 50 °C. 

**Figure 6:**
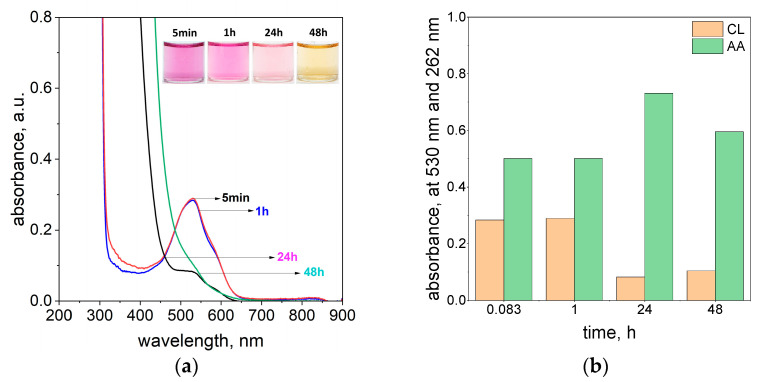
The UV-Vis spectra of the solution containing the mixture of 4 mL calcon (CL) and 0.4 g of ascorbic acid (**a**) at a higher temperature of 50 °C. The change in the absorbance value coming from CL and ascorbic acid (after 10,000 dilutions) (**b**) with time. Conditions: C_0,CL_ = 5 × 10^−5^ mol/dm^3^ (the value of concentration before mixing), T = 50 °C, and path length of 1 cm.

We started with the CL solution, for which the degradation process took 1 month at 20 °C (Supplementary Materials, Figure S4b) and determined that the observed pseudo first-rate constant equals 0.0062 h^−1^. Similar to the previous experiments, the CL solution containing 4 mL of azo dyes and 0.4 g ascorbic acid (see, Table 1) was inserted into a thermostatic bath (T = 50 °C). As can be expected the higher temperature accelerates the process of dye degradation. The intense pink color coming from the CL was quickly lost within 24 h, (Figure 6a). Next, the solution turned yellow 48 h later (Figure 6a). The characteristic UV-Vis spectra for the progressive process were recorded. The registered spectrum has a characteristic peak at a wavelength of 530 nm (Figure 6), whose intensity decreases with time. After 48 h, a complete decay of the peak was observed. 

Dilution of the sample (ten thousand times) revealed a new peak at 274 nm and spectrum lifting at 200 nm (see Supplementary Materials, Figure S10). The registered spectrum (see Supplementary Materials, Figure S10A,B) within 24 h exhibited a changed peak position towards a shorter wavelength, i.e., 262 nm (see Supplementary Materials, Figure S10C), and stronger intensity. Then, a further decrease in absorbance with time is observed (see Supplementary Materials, Figure S10D). This might suggest other mechanisms in the process of azo dye degradation. However, the appearance of the yellow color of the solution after 48 h suggests that, in the end, we yielded the same products as was the case for TR and MO.

**Figure 7:**
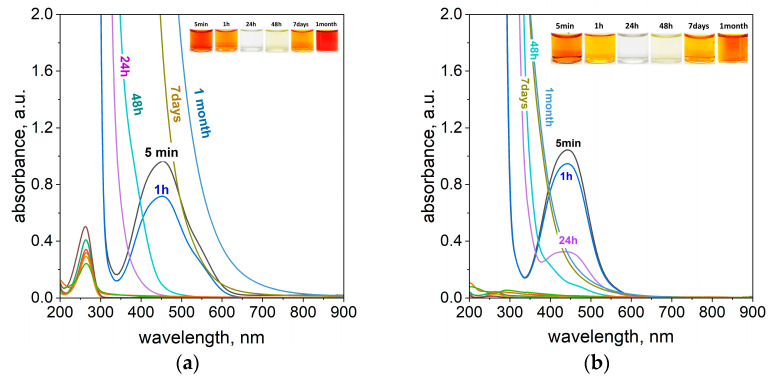

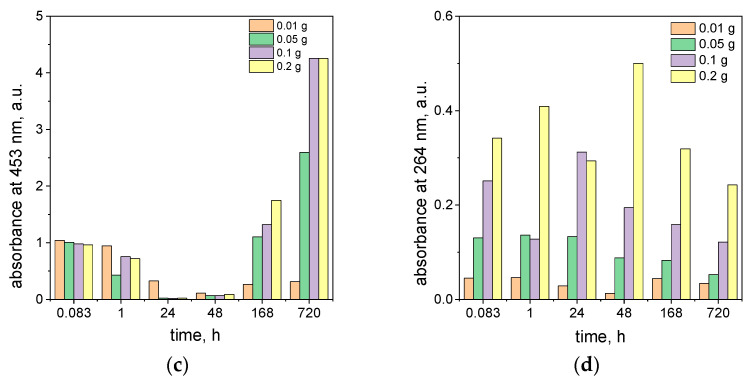
The UV-Vis spectra of the solution containing the mixture of 4 mL tropaeolin OO (TR) and ascorbic acid with different amounts of ascorbic acid: 0.2 g (**a**) and 0.01 g (**b**). The change in the absorbance value coming from TR (**c**) and ascorbic acid (after 10,000 dilutions) (**d**) with time at different initial ascorbic acid concentrations (0.01–0.2 g). Conditions: C_0,TR_ = 5 × 10^−5^ mol/dm^3^ (the value of concentration before mixing), T = 50 °C, and path length of 1 cm.

Taking into account that the process of azo dye degradation was faster for CL and the values of observed rate constants change up to 0.038 h^−1^ at 50 °C (see Supplementary Materials, Figure S11), the process of TR and MO degradation was tested for lower amounts of ascorbic acid (0.2 g–0.01 g) and high temperature. In the case of all studied TR samples, complete degradation took place within 24 h (see Figure 7a,b, Supplementary Materials, Figure S12a,b). 

For TR, the characteristic UV-Vis spectra were recorded with maxima located at 453 nm for 0.2 g of ascorbic acid, 450 nm for 0.1 g of ascorbic acid, and 445 nm for 0.05 g and 0.01 g of ascorbic acid.

Figure 7c shows that, with time, the absorbance intensity decreases, and the zero values were reached after 24 h (m_AA_ > 0.01 g). Then, a further increase in absorbance was observed at a level even higher than the initial absorbance intensity coming from the dye. This suggests that the observed changes are related to other products, which are formed during the process of azo dye degradation and were assigned to the oxidized form of ascorbic acid. The fitted curve obtained from experimental data has an exponential character (see Figure S13, Supplementary Materials), and the process of TR degradation is first order. Taking into account the huge excess of ascorbic acid compared to dye (i.e., isolation condition), the process is pseudo first order. The increase in ascorbic acid amount causes an increase in the reaction rate constant, and the values change from 0.048 to 0.325 h^−1^ for 0.2 g at 50 °C (see Supplementary Materials, Figure S13). Whereas, at 20 °C, it was 0.036 h^−1^ (see, Table 3).

The degradation of MO for the concentration of 0.2 g ascorbic acid came after 1 h (see Supplementary Materials, Figure S14a–d). However, for the lowest concentration of ascorbic acid (0.1 g–0.01 g), the color disappeared after 24 h (see Supplementary Materials, Figure S14b–d). The characteristic UV-Vis spectra were recorded with maximums for different amounts of ascorbic acid: 506 nm for 0.2 g (see Supplementary Materials, Figure S14a), 450 nm for 0.1 g (see Supplementary Materials, Figure S14b), 447 nm for 0.05 g (see Supplementary Materials, Figure S14c), and 443 nm for 0.01 g (see Supplementary Materials, Figure S14d) of ascorbic acid. As for MO, the intensity of the spectrum decreases in a short time (see Supplementary Materials, Figure S14e). For MO, similar to TR, the solutions turn yellow after 7 days. Then, they turn brown, orange, or yellow after one month, depending on the concentration of ascorbic acid. After ten thousand dilutions, the maximum (264 nm) coming from ascorbic acid was registered (see Figure 7d and Supplementary Materials, Figure S14f). Further changes in the absorbance intensity relate to the activity of ascorbic acid and other products’ appearance.

### 2.6. The Influence of Daylight Exposition on the Process of Dye Degradation

The process of azo compound degradation in the aqueous solutions was carried out in the presence of a significant excess of ascorbic acid (0.01–0.4 g) at the temperature of 20 °C. From the literature [39,40], it is known that aqueous solutions of azo compounds in the presence of catalysts are photodegraded. Therefore, the exposition of daylight on the efficiency of dye removal in the presence of ascorbic acid was also studied. Unlike in the literature, we do not use an additional compound like semiconductors (TiO_2_ or ZnO), which are usually applied [24,41,42,43,44]. During these studies, we want to verify if sunlight irradiation might have some influence on the ascorbic acid products (e.g., radicals, see [29]) and thus on the rate of azo dye degradation.

The experiments were carried out for aqueous solutions containing TR, OM, and CL. Similar to the previous study, a gradual disappearance of color was observed after mixing azo dye solutions with ascorbic acid. The rate of color degradation depends on the tested azo dyes and seems to be independent of the sunlight exposition (see, the full description of the obtained results provided in Supplementary Materials, Section S5, Figures S15–S20). To compare the rate of the process under DLP and DLE conditions, the kinetic curve for calcon was drawn (Supplementary Materials, Figure S20a). From them, the values of first-order rates were determined and were equal to 3.03 × 10^−5^ M^−1^s^−1^ (DLP) and 3.17 × 10^−5^ M^−1^s^−1^ (DLE). Thus, the calculated values of the rate constants are very close to each other.

### 2.7. The Mechanism of Azo Dye Degradation Using Ascorbic Acid

**Figure 8:**
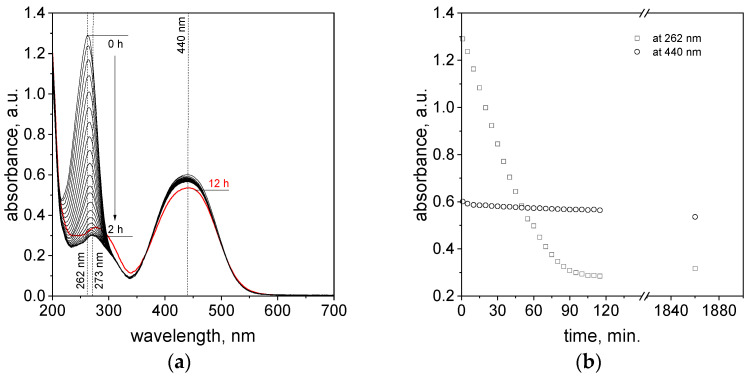
The UV-Vis spectra evolution (2 h, step of 5 min) of the solution containing the mixture of tropaeolin OO and ascorbic acid at 50 °C (**a**); Kinetic curves registered at 262 and 440 nm (**b**). Conditions: volumetric ratio of TR to ascorbic acid (1:1); the value of the concentration of tropaeolin OO after mixing with ascorbic acid: TR = 5 × 10^−5^ mol/dm^3^, and ascorbic acid = 2 × 10^−4^ mol/dm^3^.

To follow the reactions taking place between azo compounds and ascorbic acid and to propose a mechanism, the process was tracked spectrophotometrically. To register changes coming from ascorbic acid in this experiment, less excess of this compound compared to azo dyes was used. The obtained spectra evolution is shown in Figure 8.

**Figure 9:**
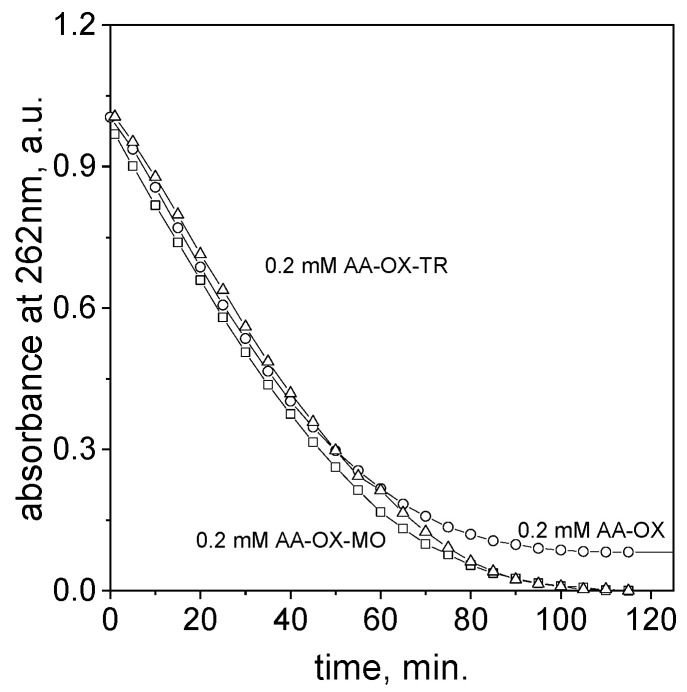
Kinetic curves registered at 50 °C for solutions containing ascorbic acid (AA-OX); ascorbic acid and MO (AA-MO); and ascorbic acid and TR (AA-TR). Conditions: volumetric ratio: 2 mL azo dyes (or water, sample AA-OX), 2 mL ascorbic acid, C_0, Azo-dyes_ = 5 × 10^−5^ mol/dm^3^, and C_0, ascorbic acid_ = 2 × 10^−4^ mol/dm^3^. Note: the values of absorbance were manipulated for AA-MO and AA-TR in such a way that the value of absorbance at 262 nm was reduced by absorbance value derived from dye (0.235 for MO and 0.285 for TR).

After reagent mixing, the characteristic spectrum with a maximum at 262 nm coming from the monoprotonated form of ascorbic acid decreases, whereas for TR, small changes were registered within 2 h (see, Figure 8b). This suggests that between reagents a reaction takes place, but it seems that one more process should be taken into account. It is known [38,45] that ascorbic acid undergoes spontaneous oxidation during air exposition. This observation is usually observed in the case of fruits, for example, the pulp of apples darkens when in contact with air. This compound also reacts with oxygen dissolved in a water-based solution, and, in consequence, it changes the reaction mechanism during the process of reduction of transition metals, e.g., Pt(IV) ions [46]. Thus, we registered the spectra evolution of ascorbic acid (with a low initial concentration, i.e., 0.2 mmol/dm^3^, which enables us to follow changes using spectrophotometric techniques) alone and with the addition of azo dyes, i.e., MO and TR. Obtained UV-Vis spectra are shown in Supplementary Materials Figure S21. Based on the obtained spectra, kinetic curves were registered and shown in Figure 9.

The character of kinetic curves in all samples are similar (Figure 9). This suggests that, in two hours, under the experimental conditions, we observe oxidation of ascorbic acid. The process follows as it is described below [47].

In aqueous solution, ascorbic acid dissociates, according to Equations (5) and (6).

(5) $$H_{2}Asc\overset{H_{2}O}{↔}H{Asc}^{-}+H^{+}$$

(6) $$HAsc^{-}\overset{H_{2}O}{↔}{Asc}^{2-}+H^{+}$$

At our reacting condition, reaction (5) takes place, and this was confirmed by the registered peak with a maximum located at 262 nm. After the dissociation process, the deprotonated form of ascorbic acid is oxidized with oxygen dissolved in water. The reaction products are free radicals, which are known from the literature and described by [47,48].

(7) $$H{Asc}^{-}+O_{2}\to {Asc}^{∙-}+{O_{2}}^{∙-}+H^{+}$$

(8) $$H{Asc}^{-}+O_{2}+H^{+}\to DHA+H_{2}O_{2}$$

The ionic oxygen radical (${O_{2}}^{∙-})$ is unstable and in an acidic environment it forms the radical ${HO}_{2}^{∙}$, according to the following reaction, known from Bielski et al. [49].

(9) $${O_{2}}^{∙-}+H^{+}↔{{HO}_{2}}^{∙}$$

Thus, the produced radical (9) and hydrogen peroxide (8) are responsible for the degradation of azo dyes. In order to confirm the presence of radicals, we performed an additional test using a known Fenton reaction. However, we applied ascorbic acid as the source of radicals instead of Fe(II) ions in the model Fenton reaction. We compared two samples. One of them contains only MO solution (orange color, pH about 5) and the second one contains a mixture of MO and ascorbic acid. To each sample, five drops of 3% solution of H_2_O_2_ were added. Then, changes in colors (from pink, Supplementary Materials, Figure S22 B, to colorless, B’) and spectra for samples containing ascorbic acid were registered (see details provided in the Supplementary Materials, Figure S22). The obtained results confirm the appearance of radicals generated during the process of ascorbic acid oxidation in the mixture solution. Finally, MO was decolorized (colorless solution, Supplementary Materials, Figure S22 sample B’). Whereas the sample containing only MO and H_2_O_2_ seems to be unchanged with time. For this sample, at a longer time, i.e., after 24 h, the spectrum was registered and compared to the initial one. Comparing these spectra, only a small change in absorbance level of 2.7% was observed. This confirms that this process is slower, and it is mostly related to the pH of the solution and the absence of radicals.

Interestingly, after 12 h, the isosbestic point at 370 nm was observed (Figure 9). It suggests that new products are formed. Taking into account that ascorbic acid oxidizes to dehydroascorbic acid (DHA), it can be expected that such a spectrum with a maximum of 185 nm and shoulders at 200 nm would be observed. Thus, this species was not confirmed in the UV-Vis study. However, considering that dehydroascorbic acid is more reactive than ascorbic acid, and at the same time it might undergo oxidation to ascorbic acid and reduction to other compounds [36], it cannot be excluded. Thus, the process is much more complex. It is also interesting that the kinetic curve (Figure 9) stops at a certain value of absorbance, whereas when azo dyes are present in the solution, the value of absorbance reaches zero. The first case can be explained by the spectra coming from products formed during ascorbic acid oxidation. The further decrease in absorbance value (see Figure S13) is related to the process of azo dye degradation. It is worth noting that ascorbic acid at small concentrations might react with azo dyes, and this process is reversible.

Whereas at higher ascorbic acid concentrations, we have a lower value of pH (see Supplementary Materials, Table S3), which positively affects the formation of radicals and hydrogen peroxide. On the other hand, the solution is a less dissociated form of ascorbic acid which is the substrate in the reactions (7) and (8).

Another approach was shown by other authors, which underlines the role of oxygen in the reacting solution. Zee et al. [50] showed that azo dyes, depending on the oxygen presence in the solution, might react in different ways. In an anaerobic solution, azo dyes undergo reduction to aromatic amines. Whereas, in the presence of oxygen, these aromatic amines undergo a rapid process of autoxidation leading to inorganic compound production.

**Figure 10:**
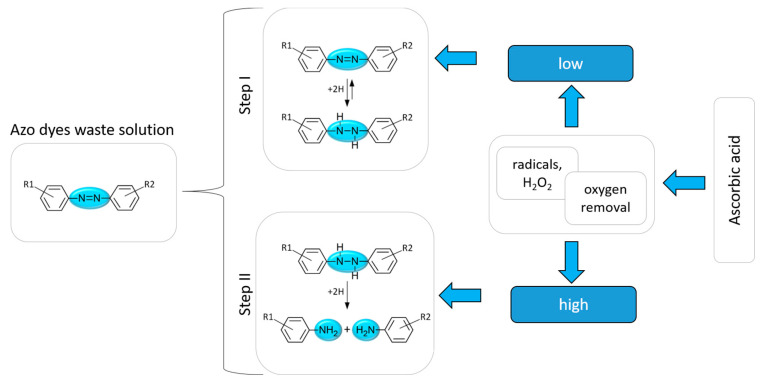
The scheme of the azo dyes’ degradation in the presence of ascorbic acid.

In this context, the addition of ascorbic acid plays a double function. The first one is related to oxygen removal from the aqueous solution and the second one relates to the azo dyes’ degradation to aromatic amines. The schematic routes are presented in Figure 10.

The addition of ascorbic acid to the solution leads to its oxidation. The consequence of this process is oxygen removal and active compound production (radicals, H_2_O_2_). Such anaerobic conditions are favorable for subsequent dye degradation steps, in which the N=N bond in the azo molecule is broken (Figure 10). In the first step, we assume the hydrogen substitution to the N=N bond, which can be reversible (Step I, Figure 10) [51,52], depending on the amount ascorbic acid added. It is in good agreement with our calculation (see Supplementary Materials, Figure S2c), which shows the spectra changes during substitution of 1 or 2 hydrogen atoms to the N=N bond.

Whereas, with a high content of ascorbic acid, step II is observed (Figure 10). For example, during the reaction of TR with ascorbic acid (high content), the peak with a maximum at about 480 nm disappears, which was confirmed with UV-Vis spectra. It is also in good agreement with the DFT calculation, which indicates that after the N=N bond breaks, the HOMO–LUMO transition is impossible (Figure S2d, Table S1, Supplementary Materials). After the reaction, each product needs two hydrogen atoms to restore the amine group. These hydrogens are in the system after oxidation of ascorbic acid. One molecule donates two hydrogen atoms, that is why we need an excess of ascorbic acid to complete the TR degradation. As a result of the N=N bond breaking, we obtain two amino products: N-phenylbenzene-1,4-diamine and 4-aminobenzenesulfonic acid. The presence of these compounds was confirmed with simulated spectra, which show that both amines absorb the light from the UV range (Figure S2c, Supplementary Materials). The degradation of calcon leads to 4-Amino-3-hydroxy-1-naphthalenesulfonic acid and 1-Amino-2-naphthol formation (see Figure S3c, Supplementary Materials). Whereas the process of methyl orange degradation using ascorbic acid produces 4-aminobenzenesulfonic acid and N,N-Dimethyl-p-phenylenediamine. In order to confirm the presence of the suggested products in the process of TR degradation, we used the LCMS technique. For this purpose, the selected samples were collected at different time points in the degradation process (5 min, 1 h, 24 h, and 1 week). Due to a huge excess of ascorbic acid, each sample was diluted 10,000 times before analysis. This approach prevents saturation of the detector and allows data to be collected for lower concentration components. The obtained results from these experiments are shown in Figure S23.

At the beginning of the process, TR (354.0906, *m*/*z*) was detected (MS spectrum shown in Figure S23, Supplementary Materials) as was ascorbic acid. With time, 24 h and 7 days later, besides ascorbic acid/dehydroascorbic acid, only one compound with a mass of 185.1073 (*m*/*z*) was detected (Figure S24). 

Further analysis (MS2, process of compound fragmentation under high-voltage condition) leads to the formation of smaller compounds; these compounds fit well to the 1,2-Diphenylhydrazine structure and confirm its presence in the solution before the MS2 approach (see Supplementary Materials, Figure S25).

Surprisingly, we do not observe other products which were suggested in the scheme (Figure 10). This could be related to the specifics of the performed experiments and the applied sample treatment before the LCMS experiments. Conditions after sample dilution are not the same as in the original sample, thus the products are different (see Figure 10, low ascorbic acid content, step I). The obtained results also drew our attention to the complexity of the process of identifying organic products and the implications related to the possible behavior of samples during dilution of the solutions. It is worth noting that the obtained product is more toxic, according to the National Library of Medicine [53] than degraded azo dye [54].

**Figure 11:**
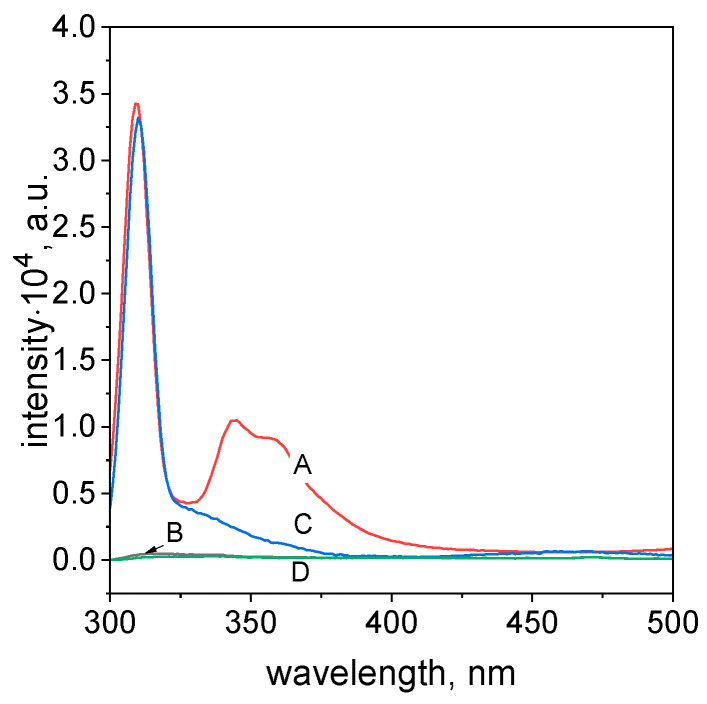
The fluorescence spectra with excitation wavelength at 280 nm obtained for ascorbic acid, tropaeolin OO, and their mixture after 5 and 60 min. Conditions: C_0,TR_= 5 × 10^−5^ mol/dm^3^ (the value of concentration before mixing), m_ascorbic acid_ = 0.2 g, and T = 50 °C. Notation: A—TR solution; B—ascorbic acid solution; C—mixture of TR and ascorbic acid, 5 min later; and D—mixture of TR and ascorbic acid, 1 h later.

Taking into account that the results obtained from LCMS are not clear, it was decided to carry out additional tests. For this purpose, a fluorescence study for the process of TR degradation was carried out. Results of excitation for TR, ascorbic acid, and TR solution containing ascorbic acid, after 5 min and 1 h, are shown in Figure 11.

Pure TR (A, Figure 11) shows a strong fluorescence signal with a max at 344 and 358 nm, whereas ascorbic acid (B, Figure 11) did not show a peak upon this excitation. After mixing both reagents together, the intensity of these two peaks that are characteristic for TR is lower (C, Figure 11) and they disappear after 1 h (D, Figure 11), confirming the azo dyes’ degradation

## 3. Materials and Methods

### 3.1. Reagents

Methyl orange (MO) (C_14_H_14_N_3_NaO_3_S, Mol. Wgt: 327.33 g/mol, p.a. Avantor Performance Materials Poland S.A., Gliwice, Poland). The base solution was prepared by dissolving 0.1 g of MO powder in 100 mL of deionized water. 

Tropaeolin OO (TR) (C_18_H_14_N_3_NaO_3_S, Mol. Wgt: 375.4 g/mol, Merck, Darmstadt, Germany). The base solution was prepared by dissolving 0.01 g of TR powder in 100 mL of deionized water. 

Calcon (CL) (C_20_H_13_N_2_NaO_5_S, Mol. Wgt: 375.40 g/mol, Merck). The base solution of CL was prepared by dissolving 0.01 g in 100 mL of deionized water.

The required concentrations of azo dye aqueous solutions (5 × 10^−5^ mol/dm^3^) were attained by diluting the appropriate volume of the stock solution with deionized water. In all experiments, the azo dye solutions were freshly prepared. The chemical structures of the used azo dyes are shown in Figure 2.

Ascorbic acid (H_2_Asc, Mol. Wgt: 176.12 g/mol p.a., Avantor Performance Materials Poland S.A.). In the experiments, the appropriate amount of L–ascorbic acid powder (0.4 g–0.01 g) was added to the 4 mL of prepared azo dye solutions.

### 3.2. Methods

UV-vis spectrophotometry. The spectra of reagents were registered using a Spectrophotometer UV-Vis (Shimadzu, Kyoto, Japan), working in the wavelength range of 190–900 nm. The solution analysis was carried out using quartz cuvettes (Hellma, Müllheim, Germany) with an optical path of 1 cm or 0.1 cm. As a reference solution, deionized water was used.

Infra-Red spectroscopy. The FTIR spectra of reagents were registered using a TENSOR II spectrophotometer (Bruker company, Ettlingen, Germany) leveraging the Attenuated Total Reflectance–Fourier transform infrared spectroscopy (ATR-FTIR) technique, using the diamond crystal. The ATR-FTIR spectra were measured in the wavenumber range 400–4000 cm^−1^ with a resolution of 1 cm^−1^ and measurements were repeated 64 times.

DFT calculations. Optimization of molecules was calculated using the DFT method with the functional B3LYP in 6–311 g base in Gaussian 16. The electronic spectra were calculated using the TD-DFT method with the same functional and base. Additionally, the CPCM solvation model with water as a solvent was used in this simulation. 

Fluorescence spectroscopy. Fluorescence spectra were measured on a spectrofluorometer FS5 (Edinburgh Instruments, Livingston, UK). The samples were measured in H_2_O solution. The excitation wavelength was set at 280 nm, and an emission in the range of 300–540 nm was observed. 

Liquid Chromatography Mass Spectrometry. The LCMS-9050 (Q-TOF), (Shimadzu, Japan). Condition of experiments: Shim-Pack Scepter C18-120 (50 mm × 2.1 mm × 3.0 µm) and reserved phase chromatography (A—water +0.1% formic acid; B—acetonitrile + 0.1% formic acid; and volume of the sample—1 µL).

## 4. Conclusions

The kinetic studies of the degradation process of azo dyes in aqueous solution using a green compound, ascorbic acid, were performed, and the mechanism was proposed. The obtained results indicate that ascorbic acid can be used in the dye removal process. However, the course of the process assumes the formation of toxic intermediates, and the process should be carried out in appropriate conditions. The obtained result shows that a high excess of this compound leads to efficient MO and TR degradation even at 20 °C within 1 to 2 days, respectively. Whereas degradation of CL must be carried out at 50 °C in order to achieve the same efficiency within 24 h. It was also shown that the process of azo dye removal is independent of the daylight exposition, and the rate of the process depends on the initial ascorbic acid concentration. For the process of dye degradation, the kinetic equation and observed rate constants were determined. The most important part of the presented study was to explain the mechanism of the azo dyes’ degradation, underlining the role of ascorbic acid as an active compound. The obtained results confirmed that ascorbic acid is responsible for oxygen removal from aqueous solution and the production of active species including ROS (HO_2_^∙^) and H_2_O_2_, which react with azo dyes leading to their degradation. The process of calcon degradation confirms that ascorbic acid is oxidized to DHA but this process is reversible. It was also shown that a high excess of ascorbic acid protects the solution over time before further aromatic amines autoxidize. Whereas ascorbic acid undergoes further oxidation, which finally leads to CO_2_ formation. Aromatic amines can be removed from waste solution via the biodegradable method or by autoxidation, but these processes occur after introducing oxygen into the solution.

## Figures and Tables

**Table 1:** The conditions of the experiments. Abbreviations: DLE—daylight exposition and DLP—daylight protection.

Initial Concentration of Azo Dye Solutions	The Initial Content of Ascorbic Acid	The Volume of Azo Dye Solution (MO, TR, CL)	T	Exposition to Daylight
C_0, TR, MO, CL_, mol/dm^3^	m_0, AA_, g	V_TR, MO, CL_, mL	°C	
The molar coefficient determination				
5 × 10^−6^		4	20	
1 × 10^−5^				
5 × 10^−5^				
1 × 10^−4^				
2 × 10^−4^				
The influence of ascorbic acid concentration and temperature on the process of dye degradation				
5 × 10^−5^	0.01	4	20	DLE
	0.05		50	
	0.10			
	0.20			
The influence of daylight exposition/protection on the process of dye degradation				
5 × 10^−5^	0.4	4	20	
			50	

**Table 2:** The values of wavelengths and molar coefficients (ε) were determined for different solutions of ascorbic acid and azo dyes, T = 20 °C. See Table 1 for detailed experimental conditions.

Solution	ɛ1 (λ1)	ɛ2 (λ2)	ɛ3 (λ3)	Ref.
	M^−1^cm^−1^	M^−1^cm^−1^	M^−1^cm^−1^	
Ascorbic acid	17,232 ± 243 (246 nm)			This work
Tropaeolin OO	12,452 ± 2 (272 nm)	25,160 ± 58 (445 nm)		[32]
Methyl Orange	14,353 ± 59 (274 nm)	39,226 ± 59 (465 nm)		[33]
Calcon	18,709 ± 20 (218 nm)	2327 ± 4 (340 nm)	6432 ± 21 (544 nm)	This work

**Table 3:** The values of observed first-order and second-order rate constants were determined at different ascorbic acid concentrations and constant temperature (20 °C).

The Concentration of Ascorbic Acid (*C_AA_*), M	Observed First-Order Rate Constant (*k_obs_*), h^−1^	
	TR	MO
0.014	0.020	0.080
0.071	0.023	0.094
0.14	0.029	0.127
0.28	0.036	0.164

## Data Availability

Data are contained within the article and Supplementary Materials.

## Supplementary Materials

The following supporting information can be downloaded at: https://www.mdpi.com/article/10.3390/molecules29153659/s1, Figure S1. The UV-Vis spectra of ascorbic acid (H_2_O as solvent) with different initial concentrations: A—1 × 10^−4^ mol/dm^3^; B—2 × 10^−4^ mol/dm^3^; C—4 × 10^−4^ mol/dm^3^; D—6 × 10^−4^ mol/dm^3^; and E—8 × 10^−4^ mol/dm^3^ (a). The dependency of absorbance (at 246 nm) vs. initial concentrations of L–ascorbic acid in the range 1 × 10^−4^ mol/dm^3^ to 8 × 10^−4^ mol/dm^3^ (b). Conditions: T = 20 °C and path length of 0.1 cm. The UV-Vis spectra for different forms of ascorbic acid calculated via the TD-DFT method (c); Figure S2. The UV-Vis spectra of tropaeolin OO aqueous solutions (H_2_O as solvent) with different initial concentrations: A—5 × 10^−6^ mol/dm^3^; B—1 × 10^−5^ mol/dm^3^; C—5 × 10^−5^ mol/dm^3^; and D—1 × 10^−4^ mol/dm^3^ (a). The dependency of absorbance vs. initial concentrations of tropaeolin OO in the concentration range from 5 × 10^−6^ mol/dm^3^ to 1 × 10^−4^ mol/dm^3^, with absorbance registered at the following wavelengths: 272 nm and 445 nm (b). Conditions: T = 20 °C and path length of 1 cm. The UV-Vis spectra for different forms of tropaeolin OO calculated via the TD-DFT method (c) and orbitals for tropaeoline OO (d). Notation: diamine = N-phenylbenzene-1,4-diamine; sulf = 4-aminobenzenesulfonic acid; tropH1 = TR with one hydrogen atom substituted to N=N; and tropH_2_ = TR with two hydrogen atoms substituted to N=N; Table S1. The calculated spectrum of tropaeolin OO has 3 maxima: 274, 331, and 480 nm; Figure S3. The UV-Vis spectra of calcon aqueous solutions (H_2_O as solvent) with different initial concentrations: A—5 × 10^−6^ mol/dm^3^; B—1 × 10^−5^ mol/dm^3^; C—5 × 10^−5^ mol/dm^3^; D—1 × 10^−4^ mol/dm^3^; and E—2 × 10^−4^ mol/dm^3^ (a). The dependency of absorbance vs. initial concentrations of calcon in the concentration range from 5 × 10^−6^ mol/dm^3^ to 2 × 10^−4^ mol/dm^3^, with absorbance registered at the following wavelengths: 218 nm, 340 nm, and 544 nm (b). Conditions: T = 20 °C and path length of 1 cm. Calculated spectra for calcon (CL) and products of its degradation: CL-amine = 1-Amino-2-naphthol and CL-sulf = 4-Amino-3-hydroxy-1-naphthalenesulfonic acid (c). Orbitals for calcon (d); Table S2. The calculated spectrum of calcon has 3 maxima: 287, 358, and 526 nm; Figure S4. The UV-Vis spectra of the solution containing the mixture of 4 mL of methyl orange (a) and calcon (b) mixing with 0.4 g of ascorbic acid; FTIR spectra for TR and ascorbic acid measured in the range 4000–400 cm^−1^ (c), and zoomed one (d–f). Conditions: C_0,MO_ = 5 × 10^−5^ mol/dm^3^ (the value of concentration before mixing), T = 20 °C, and path length of 1 cm; Table S3. The values of pH registered for the TR and AA mixture during the degradation process. Conditions: C_0, TR_ = 5 × 10^–5^ M and T = 50 °C; Figure S5. The UV-Vis spectra of the solutions containing different amounts of ascorbic acid: 0.567 mol/dm^3^ (a); 0.284 mol/dm^3^ (b); and 0.0002 mol/dm^3^ (c); solutions aged over time. Conditions: T = 50 °C and path length of 1 cm; Figure S6. The UV-Vis spectra of the solutions containing the mixture of 4 mL tropaeolin OO (TR) and ascorbic acid with different amounts of ascorbic acid: 0.1 g (a) and 0.05 g (b). The change in the absorbance value coming from TR (c) and ascorbic acid (after 10,000 dilutions) (d) with time at the different initial ascorbic acid concentrations (0.01—0.2 g). Conditions: C_0,TR_ = 5 × 10^−5^ mol/dm^3^ (the value of the concentration before mixing), T = 20 °C, and path length of 1 cm; Figure S7. The UV-Vis spectra of the solution containing the mixture of 4 mL methyl orange (MO) and ascorbic acid at different content levels: 0.2 g (a); 0.1 g (b); 0.05 g (c); and 0.01 g (d). The change in the absorbance value coming from MO (e) and ascorbic acid, (after 10,000 dilutions) (f) with time at different initial ascorbic acid concentrations (0.01—0.2 g). Note, the samples containing 0.1 and 0.2 g ascorbic acid were not analyzed after 48 h (f). Conditions: C_0,MO_ = 5 × 10^−5^ mol/dm^3^ (the value of concentration before mixing), T = 20 °C, and path length of 1 cm; Figure S8. The UV-Vis spectrum of the solution containing the mixture of 4 mL tropaeolin (TR) and ascorbic acid registered after 5 min after reagents were mixed and spectrum deconvolution. Conditions: content of ascorbic acid: 0.2 g, C_0,TR_ = 5 × 10^−5^ mol/dm^3^, T = 20 °C, and path length of 1 cm; Figure S9. The experimental data (a) and fitted kinetic curve for sample containing 0.2 g of ascorbic acid (b) for the TR solution; determination of second-order rate constant from the slope of linear fitting to experimental data for TR and MO (c). Conditions: C_0,TR_ = 5 × 10^−5^ mol/dm^3^, T = 20 °C, and path length of 1 cm; Figure S10. The spectra evolution from ascorbic acid within 3 days. Conditions: C_0,CL_ = 5 × 10^−5^ mol/dm^3^ (the value of concentration before mixing), T = 50 °C, and path length of 1 cm; Figure S11. The experimental data and fitted kinetic curves for sample containing 0.4 g of ascorbic acid solution. Conditions: C_0,CL_ = 5 × 10^−5^ mol/dm^3^, T = 50 °C, and path length of 1 cm; Figure S12. The UV-Vis spectra of solution containing the mixture of 4 mL tropaeolin OO (TR) and ascorbic acid at different concentrations of ascorbic acid: 0.2 g (a) and 0.05 g (b). Conditions: C_0,TR_ = 5 × 10^−5^ mol/dm^3^ (the value of concentration before mixing), T = 50 °C, and path length of 1 cm; Figure S13. The experimental data and fitted kinetic curves for sample containing 0.2 g of ascorbic acid solution. Conditions: C_0,TR_ = 5 × 10^−5^ mol/dm^3^, T = 50 °C, and path length of 1 cm; Figure S14. The UV-Vis spectra of solution containing the mixture of 4 mL methyl orange (MO) and ascorbic acid at different concentrations of ascorbic acid: 0.2 g (a); 0.1 g (b); 0.05 g (c); and 0.01 g (d). The change in the absorbance value coming from MO (e) and ascorbic acid (after 10,000 dilutions) (f) with time at different initial ascorbic acid concentrations (0.01—0.2 g). Conditions: C_0,MO_ = 5 × 10^−5^ mol/dm^3^ (the value of concentration before mixing), T = 50 °C, and path length of 1 cm; Figure S15. The UV-Vis spectra of the solution containing the mixture of 4 mL of tropaeolin OO (TR) and 0.4 g of ascorbic acid at different levels of exposure to daylight: daylight exposition (DLE) (a) and daylight protection (DLP) (b). Conditions: C_0,TR_ = 5 × 10^−5^ mol/dm^3^ (the value of concentration before mixing), T = 20 °C, and path length of 1 cm; Figure S16. The UV-Vis spectra of the solution containing the mixture of 4 mL of methyl orange (OM) and 0.4 g of ascorbic acid at different daylight exposure levels: daylight exposition (DLE) (a) and daylight protection (DLP) (b). Conditions: C_0,OM_ = 5 × 10^−5^ mol/dm^3^ (the value of concentration before mixing), T = 20 °C, and path length of 1 cm; Figure S17. The UV-Vis spectra of the solution containing the mixture of 4 mL of calcon (CL) and 0.4 g of ascorbic acid at different levels of exposure to daylight: daylight exposition (DLE) (a) and daylight protection (DLP) (b). Conditions: C_0,CL_ = 5 × 10^−5^ mol/dm^3^ (the value of concentration before mixing), T = 20 °C, and path length of 1 cm; Figure S18. The change in the absorbance value coming from OM, TR, and CL (a) and ascorbic acid (after 10,000 dilutions) (b) with time, in daylight protection (DLP) conditions and a constant concentration of ascorbic acid (0.4 g). Conditions: C_0,MO,TR,CL_ = 5 × 10^−5^ mol/dm^3^ (the value of concentration before mixing), T = 20 °C, and path length of 1 cm; Figure S19. The UV-Vis spectra of the solution containing the mixture of 4 mL of calcon (CL) and 0.4 g of ascorbic acid at different levels of exposure to daylight: daylight exposition (DLE) (a) and daylight protection (DLP) (b). Conditions: C_0,CL_ = 5 × 10^−5^ mol/dm^3^ (the value of concentration before mixing), T = 20 °C, and path length of 1 cm; Figure S20. The kinetic curves registered for calcon at 530 nm (a) with fitting equation to obtained experimental data for DLE and DLP sample. The kinetic curves registered at 272 nm (b) for DLE and DLP sample. Conditions: m_ascorbic acid_ = 0.4 g (0.57 mol/dm^3^), C_0,CL_ = 5 × 10^−5^ mol/dm^3^, T = 20 °C, and path length of 1 cm; Figure S21. The UV-Vis spectra evolution (2 h, step 5 min) registered at 50 °C for solutions containing ascorbic acid (AA-OX) (a) and ascorbic acid and MO (AA-MO) (b). Conditions: volumetric ratio: 2 mL MO (or water): 2 mL ascorbic acid, C_0, MO_ = 5 × 10^−5^ mol/dm^3^, and C_0, ascorbic acid_ = 2 × 10^−4^ mol/dm^3^; Figure S22. A—sample containing MO and H_2_O_2_ (t = 2 min); A’—sample containing MO and H_2_O_2_ (t = 30 min); B—sample containing MO, AA, and H_2_O_2_ (t = 2 min); and B’—sample containing MO, AA, and H_2_O_2_ (t = 30 min). Conditions: C_0, MO_ = 5 × 10^−5^ mol/dm^3^, m_AA_ = 0.025 g, V_MO_ = 4 mL, and T = 20 °C; Figure S23. MS spectrum of TR solution containing ascorbic acid after 5 min. Conditions before dilution: C_0, TR_ = 5 × 10^−5^ mol/dm^3^, m_ascorbic acid_ = 0.2 g, and T = 20 °C. Sample was diluted before experiment 10,000 times; Figure S24. The MS spectrum of the TR degradation product (7 days later); Figure S25. The MS2 spectrum (after fragmentation).
