# Supplementary material for: The Role of Ascorbic Acid in the Process of Azo Dye Degradation in Aqueous Solution
Source: Molecules. 2024 Aug 2;29(15):3659. doi: 10.3390/molecules29153659 (PMC11313820; doi:10.3390/molecules29153659)
Supplement: Supplementary file 1 [file molecules-29-03659-s001.zip › molecules-3112207-supplementary.pdf]

Supporting Materials

The role of ascorbic acid in the process of azo dyes degradation in aqueous solution

Adrianna Pach[1], Aleksandra Zaryczny[1], Agnieszka Podborska[2] and Magdalena Luty-Błocho[1,*]

[1] AGH University of Krakow, Faculty of Non-Ferrous Metals, al. Adama Mickiewicza 30, 30-059 Krakow, Poland

[2] AGH University of Krakow, Academic Centre for Materials and Nanotechnology, al. Adama Mickiewicza 30, 30-059 Krakow, Poland; podborsk@agh.edu.pl

*Corresponding author: mlb@agh.edu.pl

S1. Spectra of reagents and molar coefficient determination

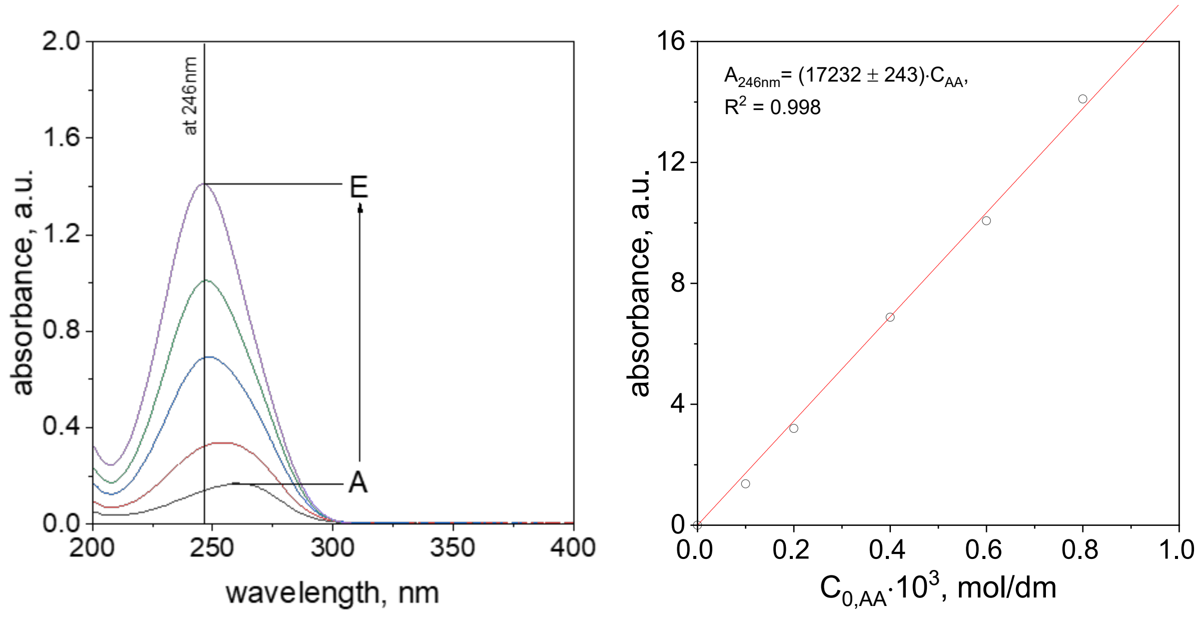

(a) (b)

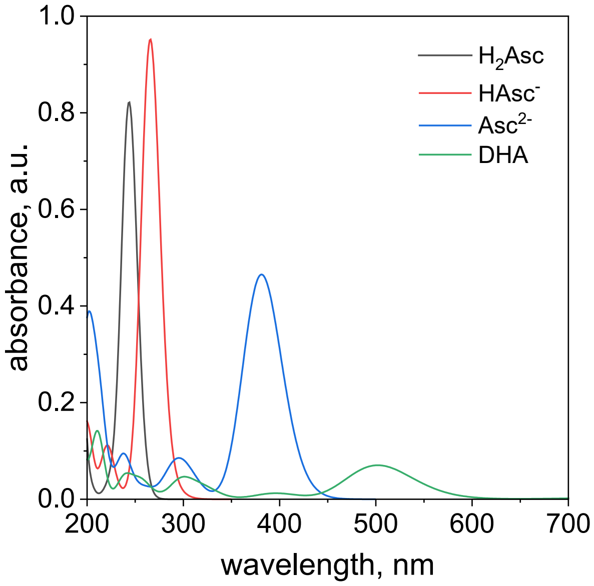

(c)

Figure S1. The UV-Vis spectra of ascorbic acid (H_2O as solvent) with different initial concentrations: A – 1· 10^{-4} mol/dm^3; B – 2· 10^{-4} mol/dm^3; C – 4· 10^{-4} mol/dm^3; D – 6· 10^{-4} mol/dm^3; E – 8· 10^{-4} mol/dm^3 (a); The dependency of absorbance (at 246 nm) vs. initial concentrations of L - ascorbic acid in the range 1· 10^{-4} mol/dm^3 to 8· 10^{-4} mol/dm^3 (b). Conditions: T = 20°C, path length 0.1 cm; The UV-Vis spectra for different form of ascorbic acid calculated by TD-DFT method (c).

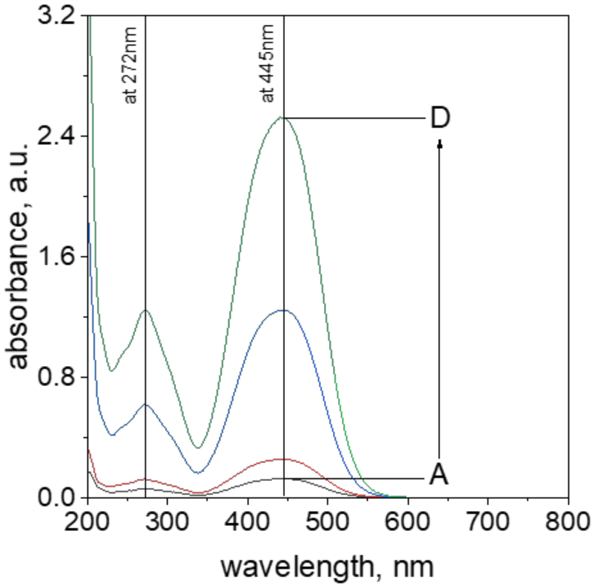

(a)

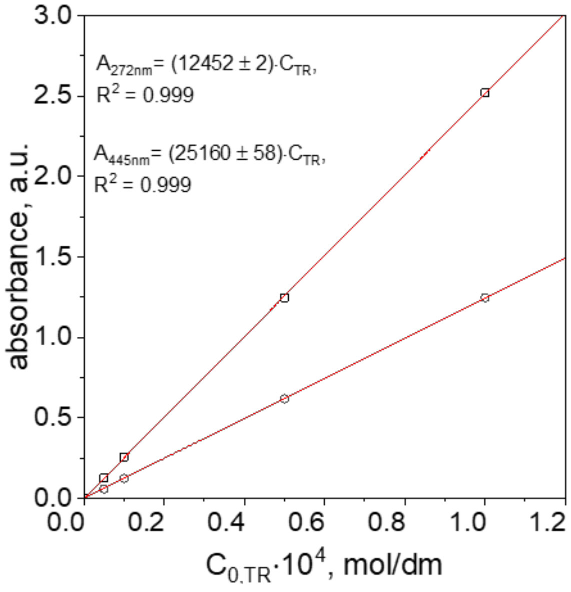

(b)

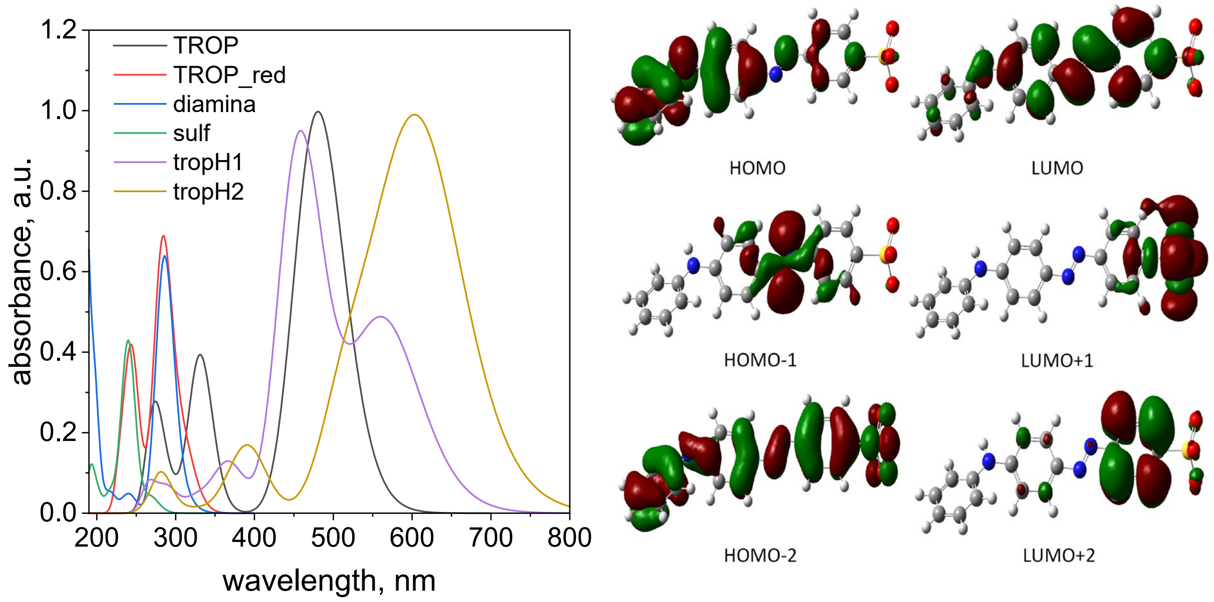

(c) (d)

Figure S2. The spectra UV-Vis of tropaeolin OO aqueous solutions (H_2O as solvent) with different initial concentrations: A – 5· 10^{-6} mol/dm^3; B – 1· 10^{-5} mol/dm^3; C – 5· 10^{-5} mol/dm^3; D – 1· 10^{-4} mol/dm^3 (a); The dependency of absorbance vs. initial concentrations of tropaeolin OO in the concentration range from 5· 10^{-6} mol/dm^3 to 1· 10^{-4} mol/dm^3, absorbance registered at wavelengths: 272 nm and 445 nm (b). Conditions: T = 20°C, path length 1 cm. The UV-Vis spectra for different forms of tropaeolin OO calculated by TD-DFT method (c); Orbitals for tropaeoline OO (d). Notation: diamine = N-phenylbenzene-1,4-diamine; sulf = 4-aminobenzenesulfonic acid; tropH1 = TR with one hydrogen atom substituted to N=N; tropH$_2$ = TR with two hydrogen atoms substituted to N=N.

Table S1. The calculated spectrum of tropaeolin OO has 3 maxima: 274, 331, and 480 nm.

Maximum	λ1	λ2	λ3
Wavelength	274 nm	331 nm	480 nm
Electron transition	HOMO-LUMO+2	HOMO-2 - LUMO	HOMO - LUMO

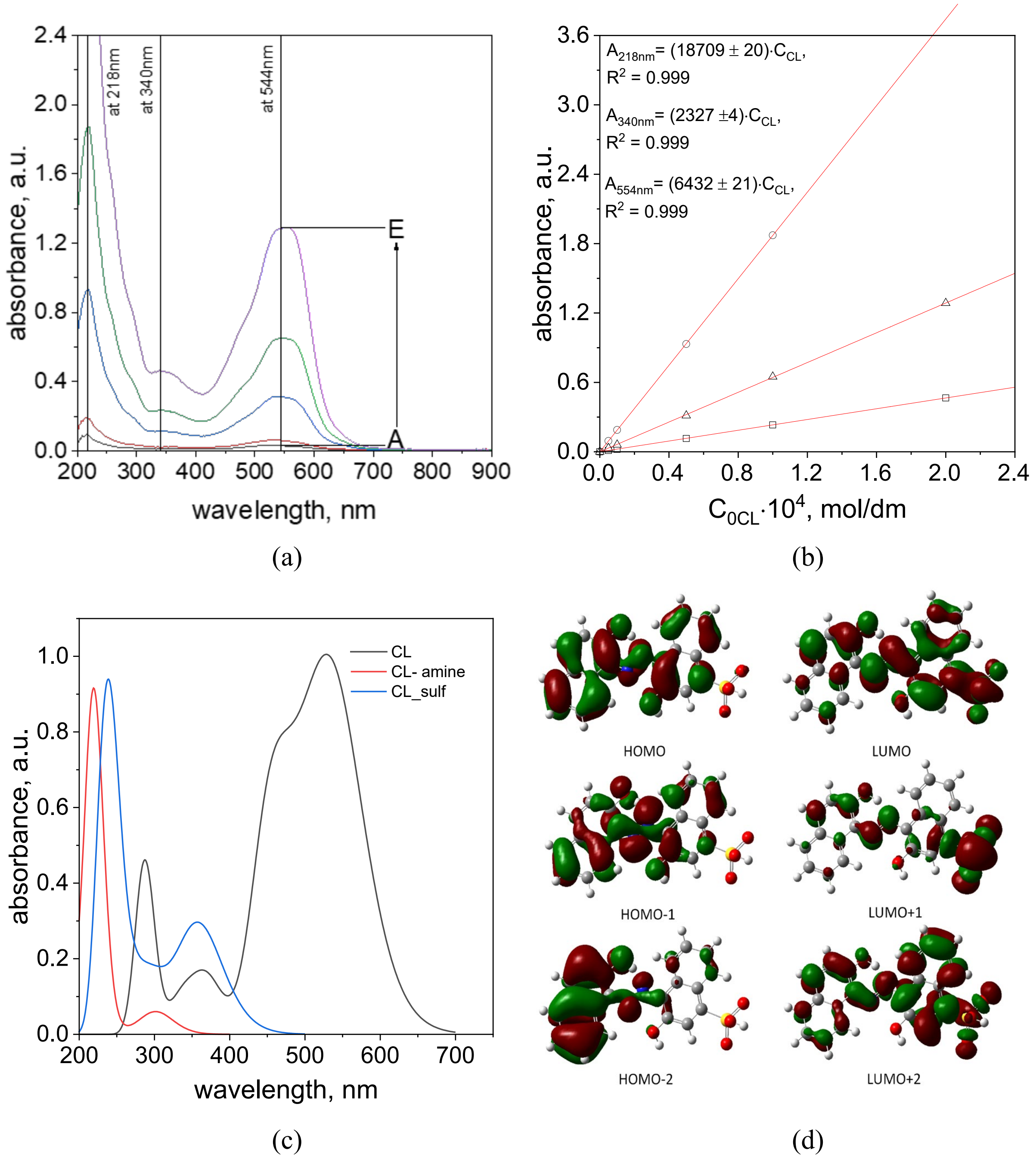

Figure S3. The spectra UV-Vis of calcon aqueous solutions (H_2O as solvent) with different initial concentrations: A – 5· 10^{-6} mol/dm^3; B – 1· 10^{-5} mol/dm^3; C – 5· 10^{-5} mol/dm^3; D – 1· 10^{-4} mol/dm^3; E – 2· 10^{-4} mol/dm^3 (a). The dependency of absorbance vs. initial concentrations of calcon in the concentration range from_5· 10^{-6} mol/dm^3 to 2· 10^{-4} mol/dm^3, absorbance registered at wavelengths: 218 nm, 340 nm and 544 nm (b). Conditions: T = 20°C, path length 1 cm. Calculated spectra for calcon (CL) and products of its degradation: CL-amine = 1-Amino-2-naphthol, CL-sulf = 4-Amino-3-hydroxy-1-naphthalenesulfonic acid (c); Orbitals for calcon (d).

Table S2. The calculated spectrum of calcon has 3 maxima: 287, 358, and 526 nm.

Maximum	λ1	λ2	λ3
Wavelength	287 nm	358 nm	526 nm
Electron transition	HOMO-LUMO+3	HOMO– LUMO+1	HOMO - LUMO

S2. The process of azo dyes degradation

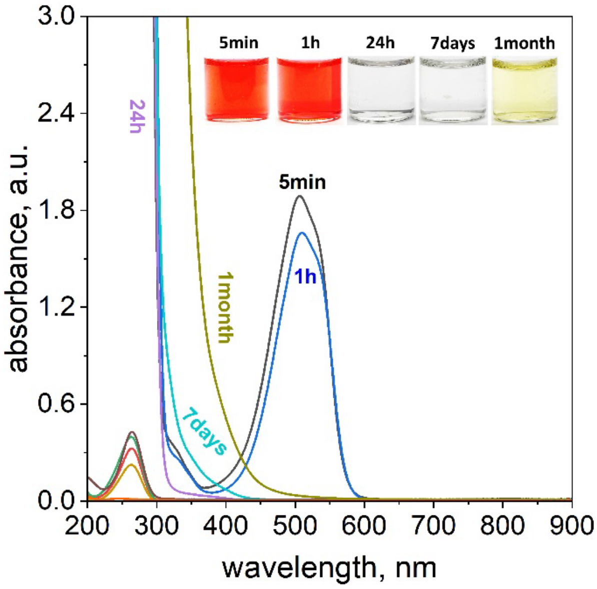

(a)

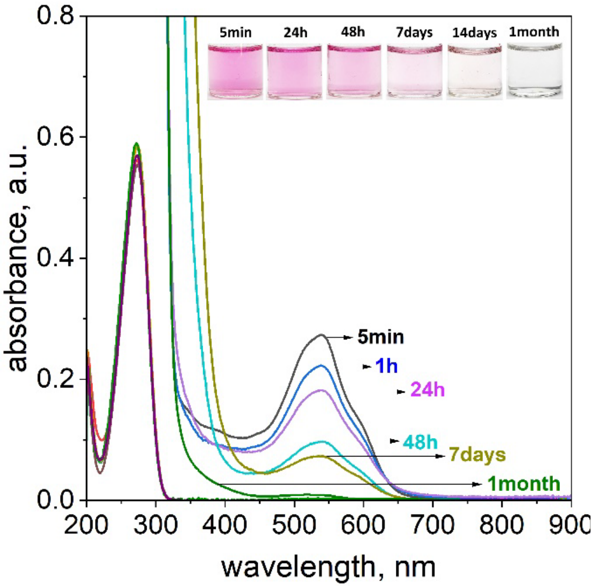

(b)

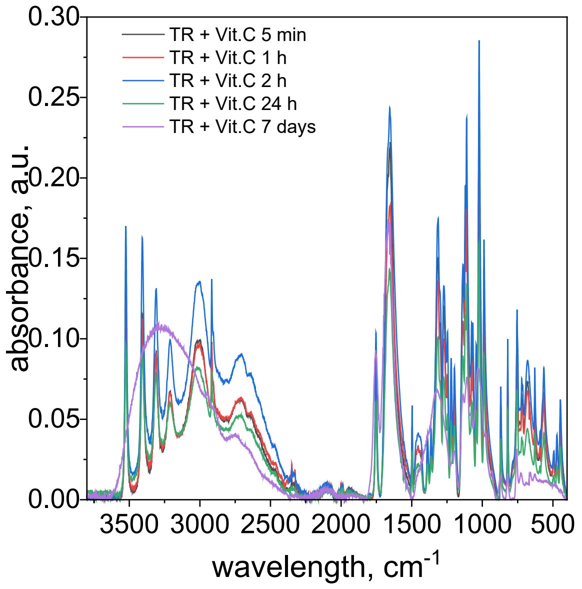

(c)

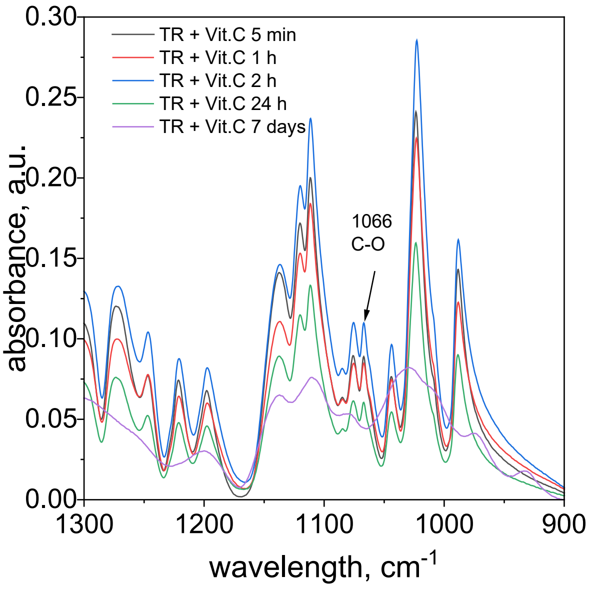

(d)

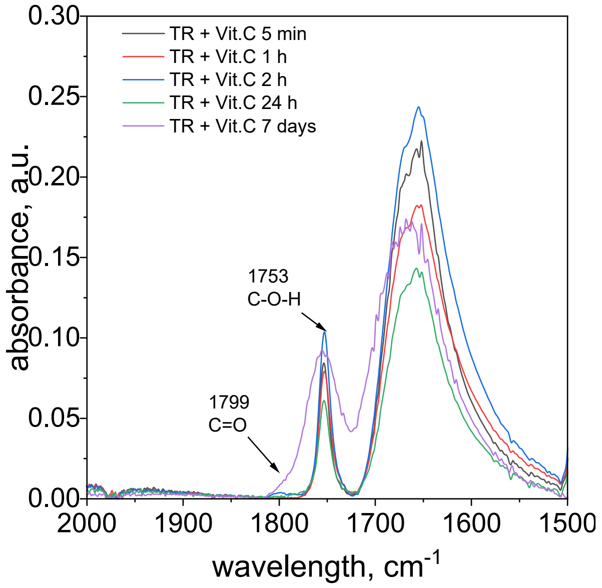

(e)

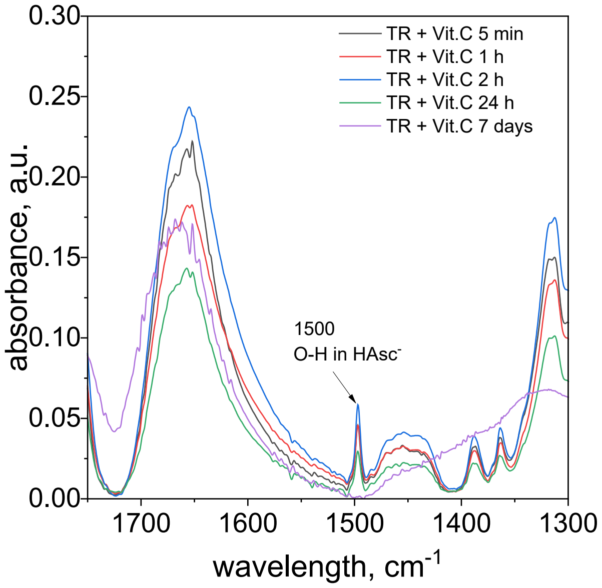

(f)

Figure S4. The UV-Vis spectra of the solution containing the mixture of 4 mL of methyl orange (a) and calcon (b) mixing with 0.4 g of ascorbic acid (Vit. C); FTIR spectra for TR and ascorbic acid (Vit. C) measured in the: 4000-400 cm^{-1} (c), and zoomed one (d)-(f). Conditions: $C_{0,MO} = 5\cdot 10^{-5}$ mol/dm^3 (the value of concentration before mixing), T = 20°C, path length 1 cm.

Table S3. The values of pH registered for TR and AA mixture during degradation process. Conditions: $C_{0,\,TR} = 5\cdot 10^{-5}$ M, T = 50°C.

Amount of AA	pH of the solution registered during degradation process			
	5 min.	1 h	24 h	7 days
0.4 g	2.14	2.08	2.12	2.23
0.01g	2.42	2.33	2.35	2.59

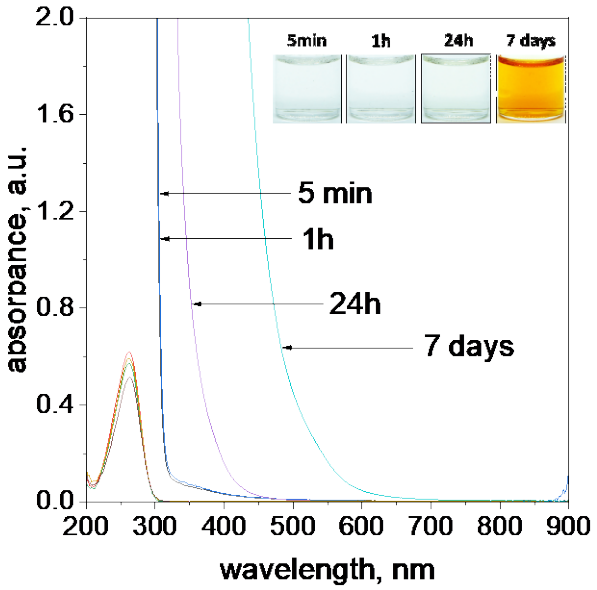

(a)

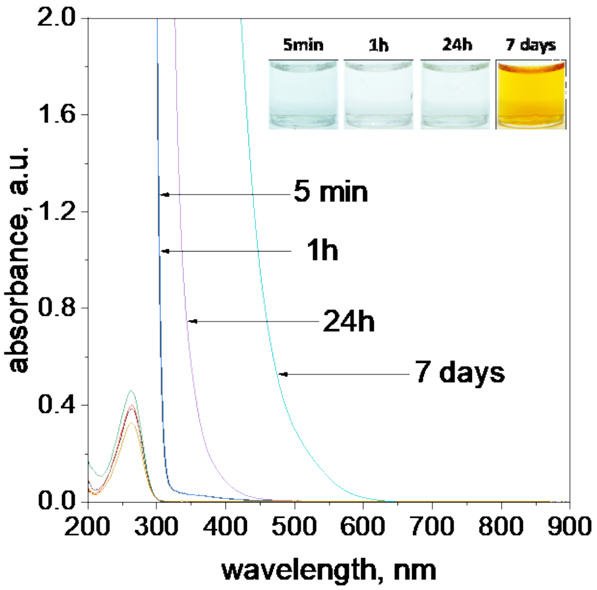

(b)

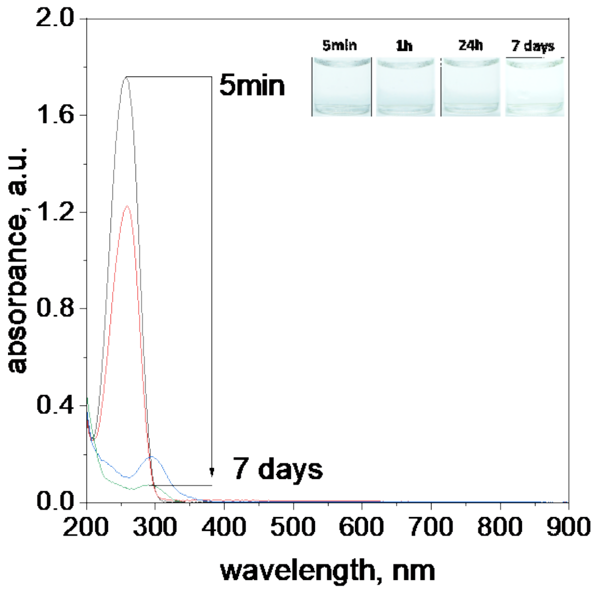

(c)

Figure S5. The UV-Vis spectra of solution containing the different content of ascorbic acid: 0.567 mol/dm^3 (a); 0.284 mol/dm^3 (b) and 0.0002 mol/dm^3 (c), solutions aged over time. Conditions: T = 50°C, path length 1 cm. Spectra with smaller intensity, registered in the range 200 – 300 nm, were collected after 10,000 times dilution (a, b).

S3. The influence of the ascorbic acid concentration on the process of azo dyes degradation

Degradation of tropeaolin OO using ascorbic acid

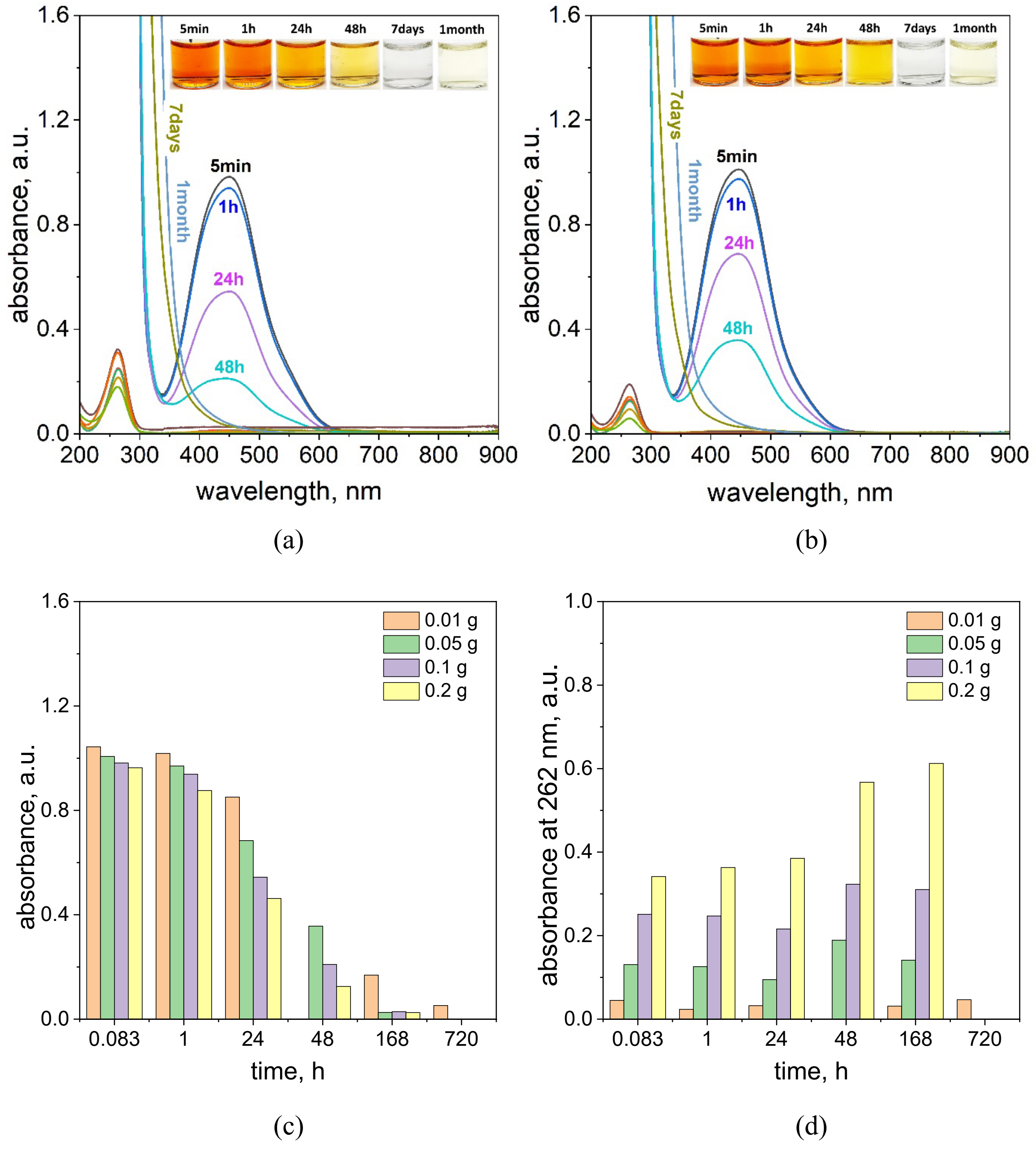

Figure S6. The UV-Vis spectra of solution containing the mixture of 4mL tropaeolin OO (TR) and ascorbic acid at different contents of ascorbic acid : 0.1 g (a); 0.05 g (b). The change of the absorbance value coming from TR (c) and ascorbic acid (after 10,000 times dilution) (d) with time at the different initial ascorbic acid concentrations (0.01 – 0.2 g). Conditions: $C_{0,TR} = 5 \cdot 10^{-5}$ mol/dm^3 (the value of concentration before mixing), T = 20°C, path length 1 cm.

Degradation of methyl orange using ascorbic acid

The registered color changes and registered spectra evolution for MO were shown in Fig. S7a-d. The process of dyes degradation at different ascorbic acid content (0.2 g, 0.1 g, 0.05 g, 0.01 g) for aqueous solutions of MO was faster than for the corresponding experiment with TR. The color of samples for 0.2 g and 0.1g of ascorbic acid changed from intensive red (5 min, 1 h) to colorless after 24 h, (see, Fig. S7a,b). At a ascorbic acid content of 0.05 g, the solution turns pale pink after 24 h, while the color disappears after 48 h (Fig. S7c). For the lowest ascorbic acid content (0.01 g), color degradation occurs after 7 days, (Fig. S7d). For all samples, after 1 month, the color of the solutions turned yellow. The UV–Vis spectra for the obtained solutions containing 0.2 g, 0.1 g and 0.05 g have maximum localized at wavelength 506 nm (see, Fig. S7a-c). Whereas the registered spectrum for the sample with 0.01 g of ascorbic acid has a maximum wavelength 502 nm, (see, Fig. S7d). The decrease in the intensity of the spectra was dependent on the amount of added Vitamin C. In the case of 0.2 g and 0.1 g amounts of ascorbic acid, the overall decrease in spectrum intensity after 24 h, was registered. For 0.05 g and 0.01 g of ascorbic acid peaks disappeared after 48 h for 0.05 g (see, Fig. S7b) and after 7 days for 0.01 g (Fig. S7d). The registered small value of the absorbance intensity, which increases after 168 h (see, Fig. S7a-d) is related rather to other processes as it was suggested in paragraph 3.2. On account of the high concentration of ascorbic acid in the solution, the samples were diluted ten thousand times to observe the spectrum coming from ascorbic acid and possible changes (Fig. S7f). With time, we observed that the value of absorbance for ascorbic acid slightly changed with time (Fig. S7f).

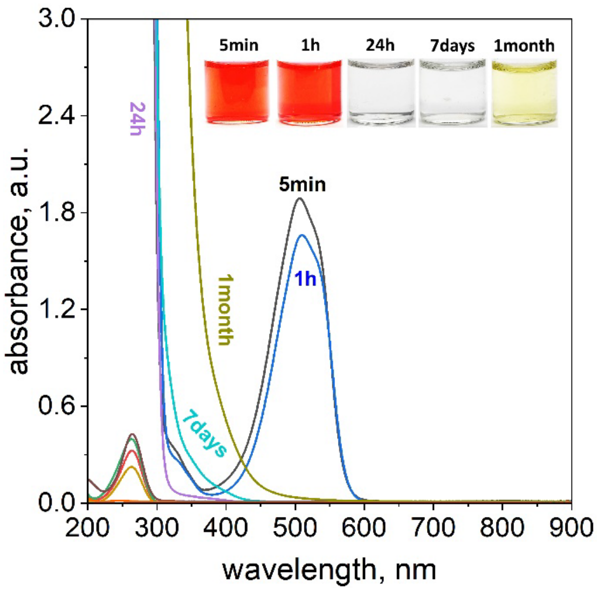

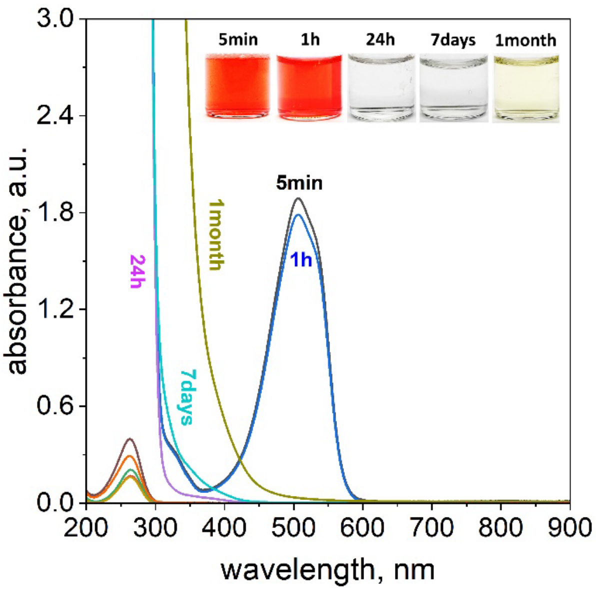

(a) (b)

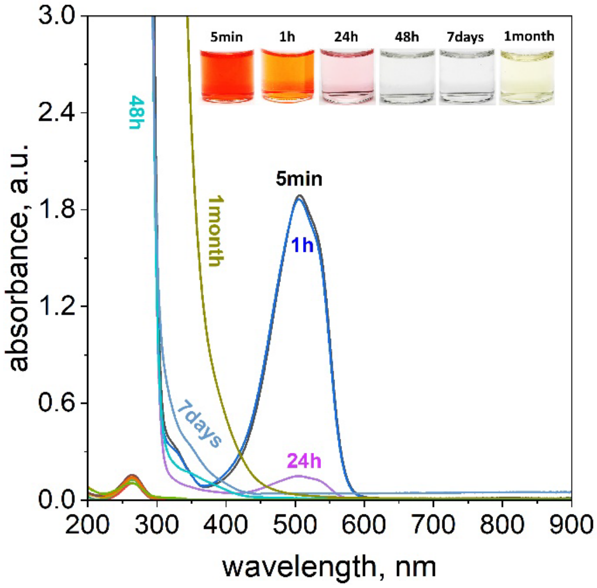

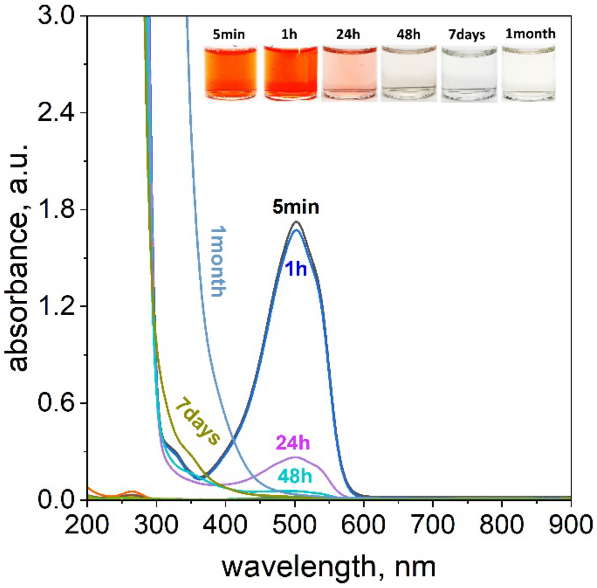

(c) (d)

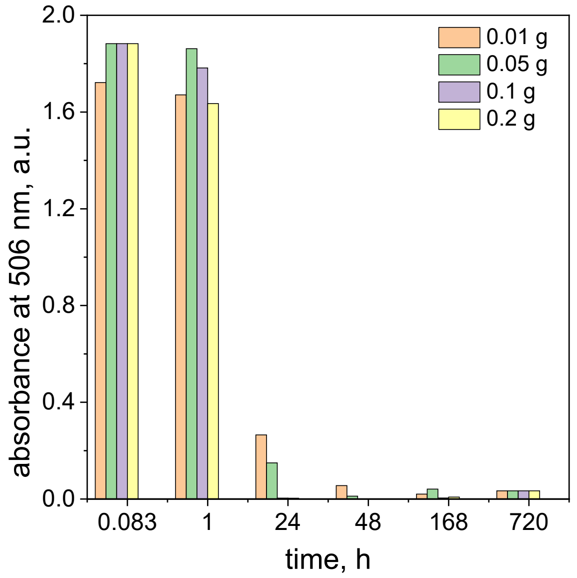

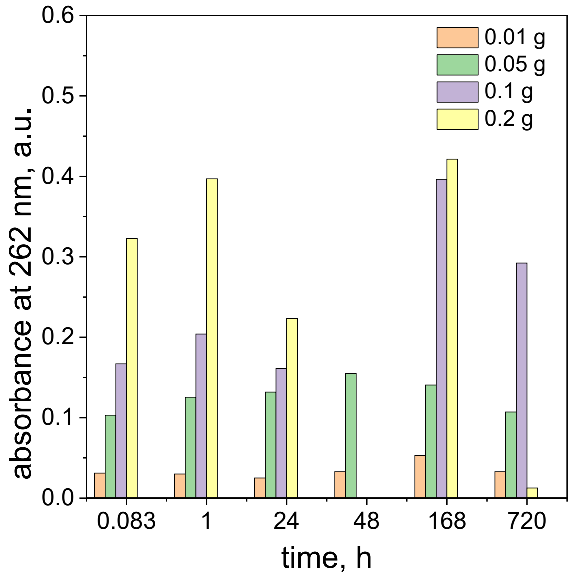

(e) (f)

Figure S7. The UV-Vis spectra of solution containing the mixture of 4 mL methyl orange (MO) and ascorbic acid at different content: 0.2 g (a); 0.1g (b); 0.05g (c) 0.01 g (d). The change of the absorbance value coming from MO (e) and ascorbic acid, (after 10,000 times dilution) (f) with time at different initial ascorbic acid concentration (0.01 – 0.2 g). Note, the samples containing 0.1 and 0.2 g Vitamin C were not analyzed after 48 h (f). Conditions: $C_{0,MO} = 5\cdot 10^{-5}$ mol/dm^3 (the value of concentration before mixing), T = 20°C, path length 1 cm.

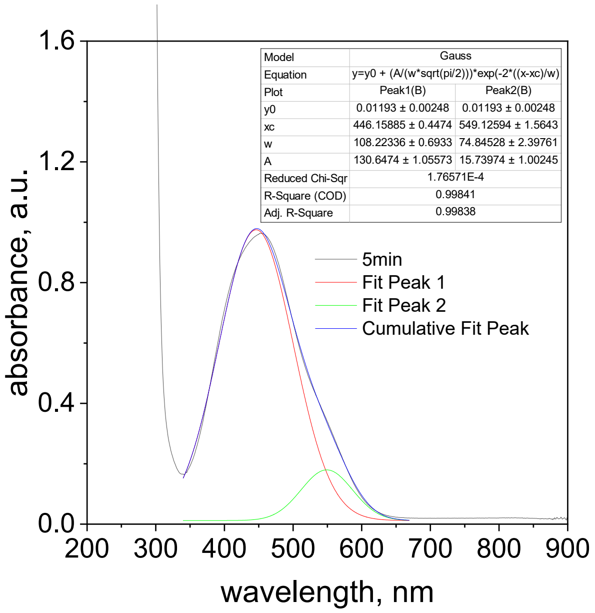

Figure S8. The UV-Vis spectrum of the solution containing the mixture of 4 mL tropaeolin (TR) and ascorbic acid registered after 5 min. after reagents were mixed and spectrum deconvolution. Conditions: content of ascorbic acid: 0.2 g, $C_{0,TR} = 5\cdot 10^{-5}$ mol/dm^3, T = 20°C, path length 1 cm.

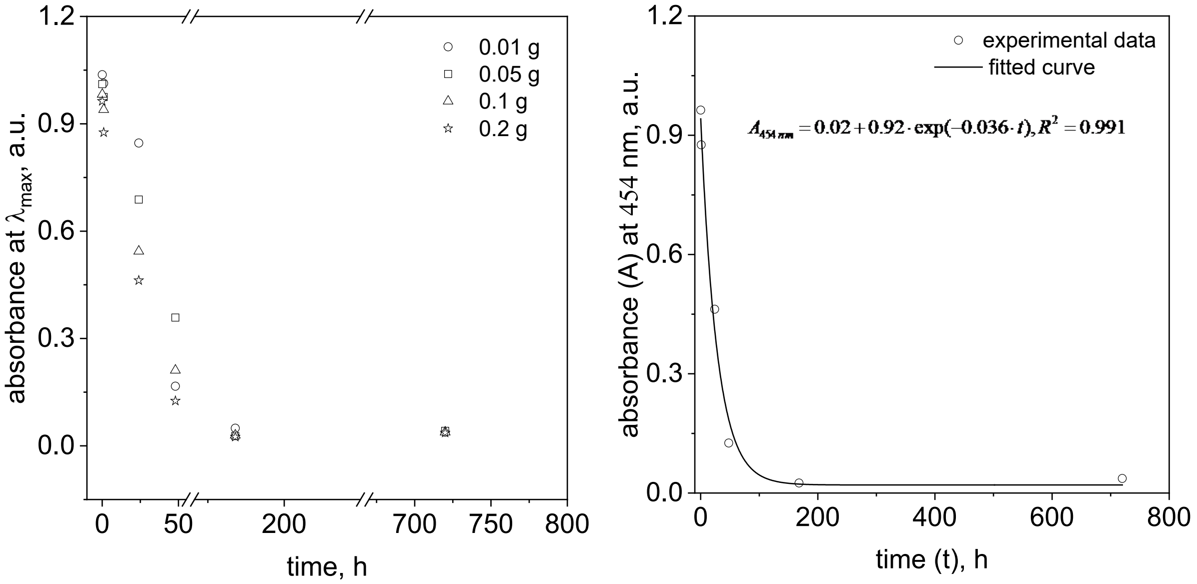

(a) (b)

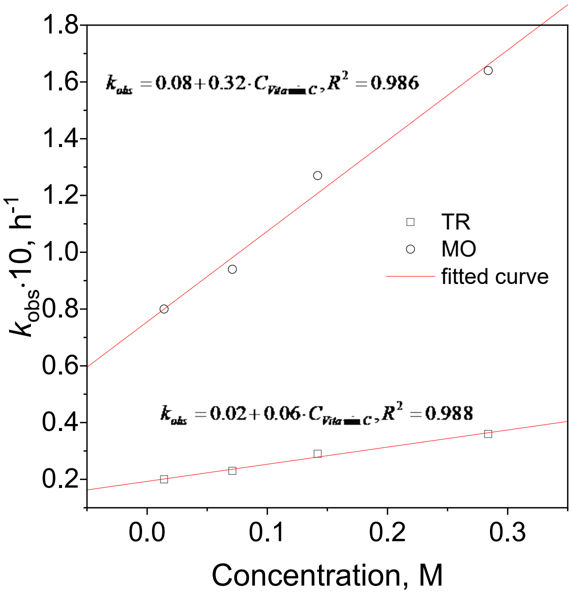

(c)

Figure S9. The experimental data (a) and fitted kinetic curve for sample containing 0.2 g of ascorbic acid (b) for the TR solution; determination of second – order rate constant from the slope of linear fitting to experimental data for TR and MO (c). Conditions: $C_{0,TR} = 5 \cdot 10^{-5}$ mol/dm^3, T = 20°C, path length 1 cm.

S4. The influence of temperature on the process of dyes degradation

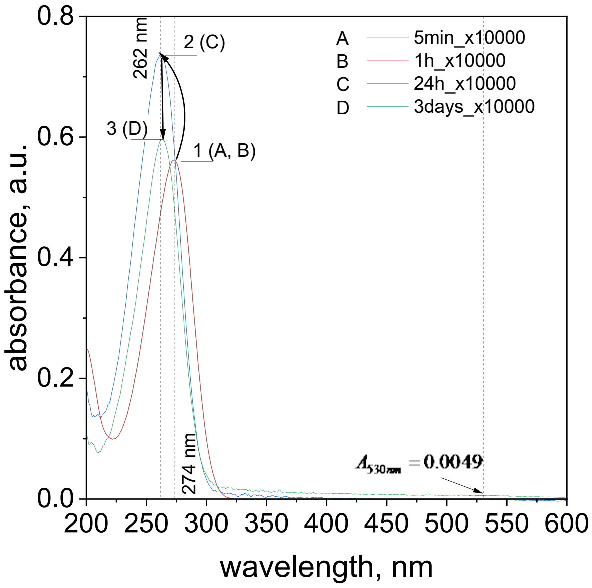

Figure S10. The spectra evolution from ascorbic acid within 3 days. Conditions: $C_{0,CL} = 5 \cdot 10^{-5}$ mol/dm^3 (the value of concentration before mixing), T = 50°C, path length 1 cm.

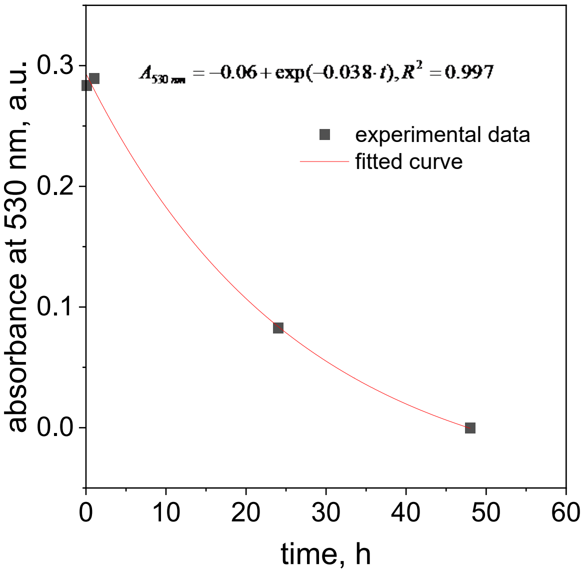

Figure S11. The experimental data and fitted kinetic curves for sample containing 0.4 g of ascorbic acid solution. Conditions: $C_{0,CL} = 5 \cdot 10^{-5}$ mol/dm^3, T = 50°C, path length 1 cm.

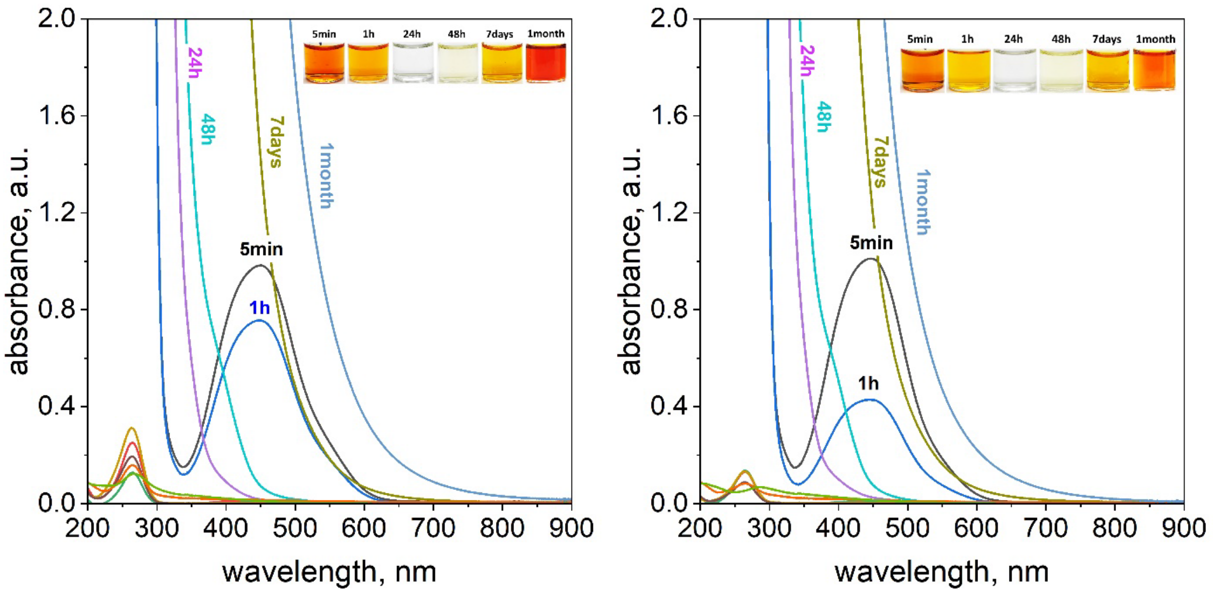

(a) (b)

Figure S12. The UV-Vis spectra of solution containing the mixture of 4mL tropaeolin OO (TR) and ascorbic acid at different contents of ascorbic acid: 0.1 g (a); 0.05 g (b). Conditions: $C_{0,TR} = 5 \cdot 10^{-5}$ mol/dm^3 (the value of concentration before mixing), T = 50°C, path length 1 cm.

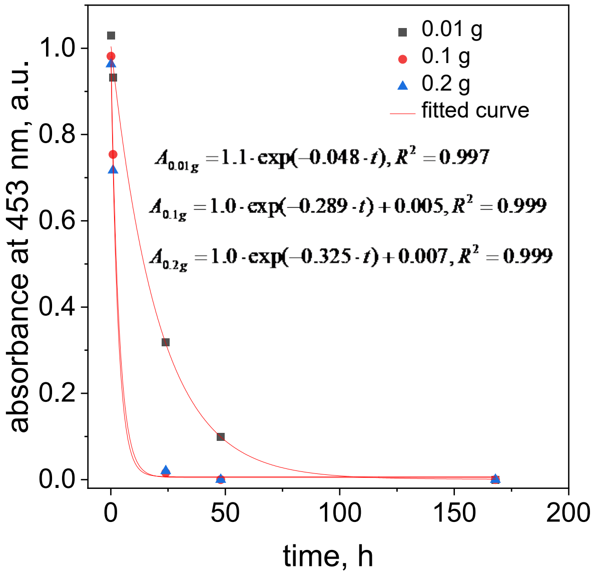

Figure S13. The experimental data and fitted kinetic curves for sample containing 0.2 g of ascorbic acid solution. Conditions: $C_{0,TR} = 5 \cdot 10^{-5}$ mol/dm^3, T = 50°C, path length 1 cm.

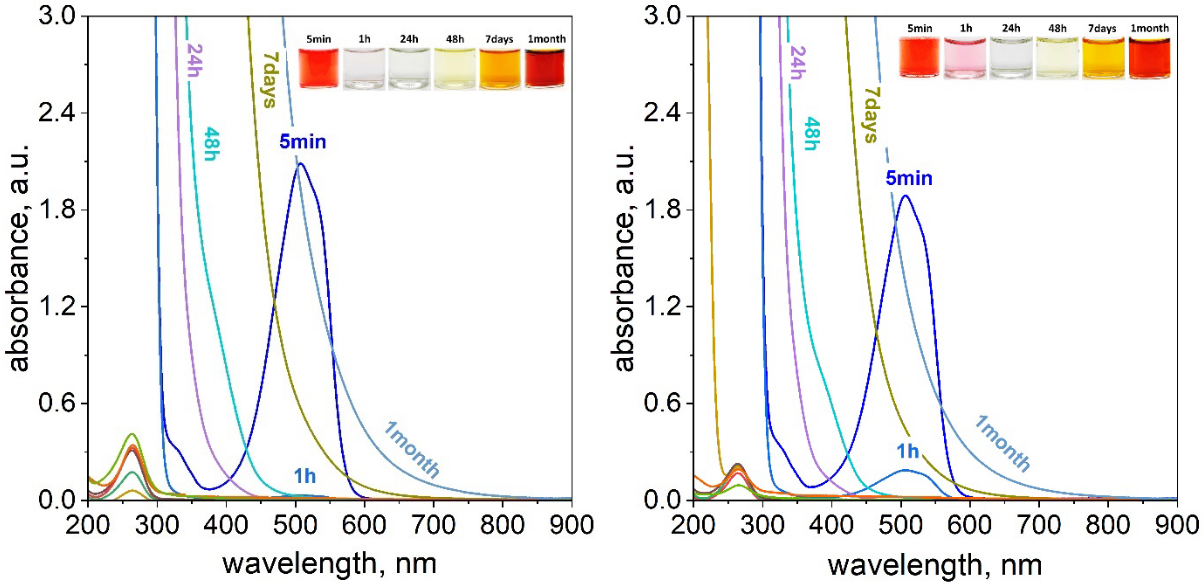

(a) (b)

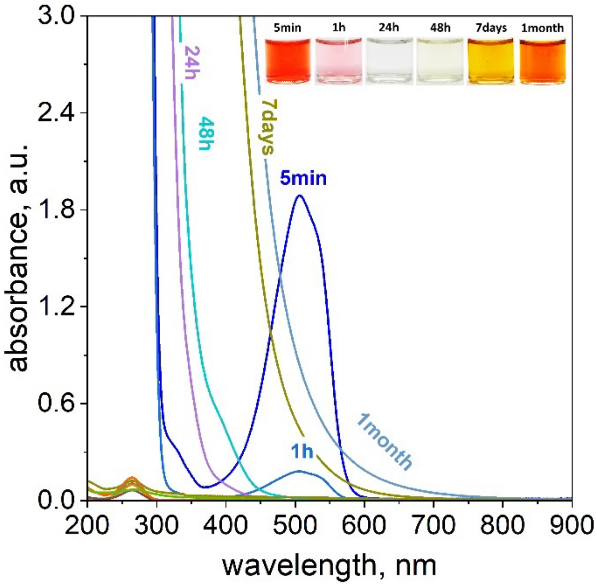

(c)

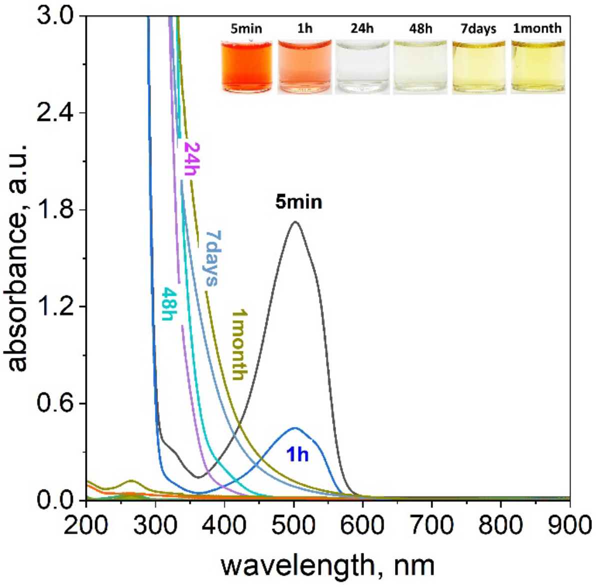

(d)

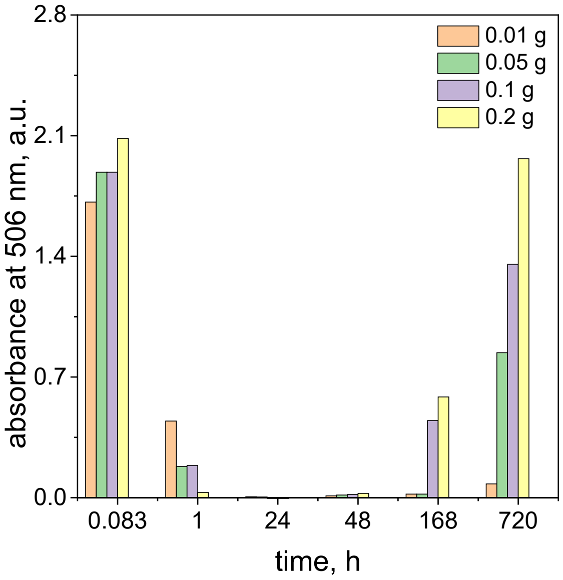

(e)

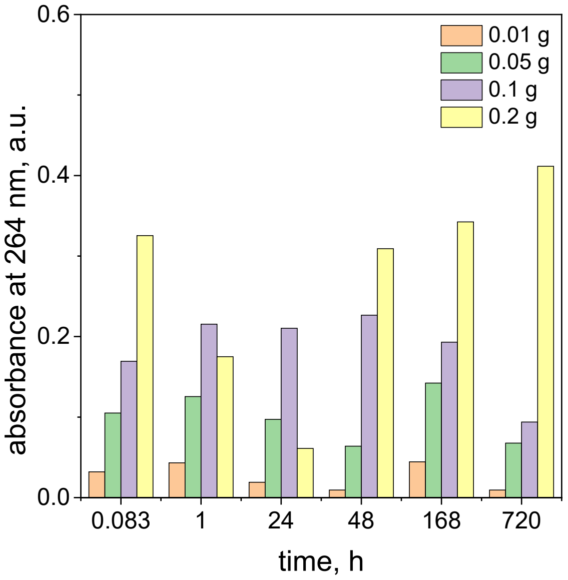

(f)

Figure S14. The UV-Vis spectra of solution containing the mixture of 4mL methyl orange (MO) and ascorbic acid at different content of ascorbic acid: 0.1 g (a); 0.2g (b); 0.05g (c) 0.01 g (d). The change of the absorbance value coming from MO (e) and ascorbic acid (after 10,000 times dilution) (f) with time at different initial ascorbic acid concentration (0.01 – 0.2 g). Conditions: $C_{0,MO} = 5 \cdot 10^{-5}$ mol/dm^3 (the value of concentration before mixing), T = 50°C, path length 1 cm.

S5. The influence of daylight exposition on the process of dyes degradation

In the process of TR degradation, the orange color coming from TR fades within 24 h and completely disappears after 48 h both for DLE (see Fig. S15a SM) and DLP (see Fig. S15b SM)

samples. The registered UV-Vis spectrum with maximum localized at wavelength 460 nm decreases over time. In the case of TR registered subtle differences in the spectra between samples exposed to sunlight (DLE) and protected from light (DLP). The intensity of UV – Vis spectra for the DLP sample was higher than for the DLE after 1 h and 24 h (SM, Fig. S15a,b). This confirms the sensitivity of aqueous azo dye solutions to sunlight. The samples were analyzed after 1 month.

In the case of MO for both DLE (see SM Fig. S16a) and DLP (see SM fig. S16b) samples obtained UV –Vis spectra after 5 min have one characteristic maximum at 510 nm, with their intensity decreasing at the same rate for both samples (1 h, 24 h).The registered spectrum from MO completely disappears for sample DLE/DLP after 24 h (SM, Fig. S16a,b).

The samples containing TR and OM turn yellow one month later (both DLE and DLP samples). As in the previous case, this may be related to the oxidation of ascorbic acid.

The process of MO and TR degradation occurs relatively faster than for the solution containing calcon. The pink color coming from the CL disappears 1 month later (SM, Fig. S17a,b). For this dye, the registered spectrum has one maximum localized at a wavelength of about 530 nm and its intensity decreases over time. In the case of CL, the effect of sunlight was more pronounced than for tropaeolin OO and methyl orange (SM, Fig. S15, S16). The evolution spectra registered for DLP sample was slower compared to the DLE sample later (SM, Fig. S17a,b and S18).

After ten-thousand-fold dilution of the samples, the location of the characteristic maximum derived from ascorbic acid, was identified. However, the recorded spectrum has a maximum wavelength of about 272 nm and it is different from that obtained in the experiments with TR and MO (see, SM Fig. S15 and S16). It might suggest that during reactions between calcon and ascorbic acid different products are formed. Interesting is also strong blue shift of the spectrum shoulder (grey area, SM Fig. S19a) and isosbestic point located at 432 nm (see SM fig. S19a-c), which again suggests different products during oxidation of ascorbic acid.

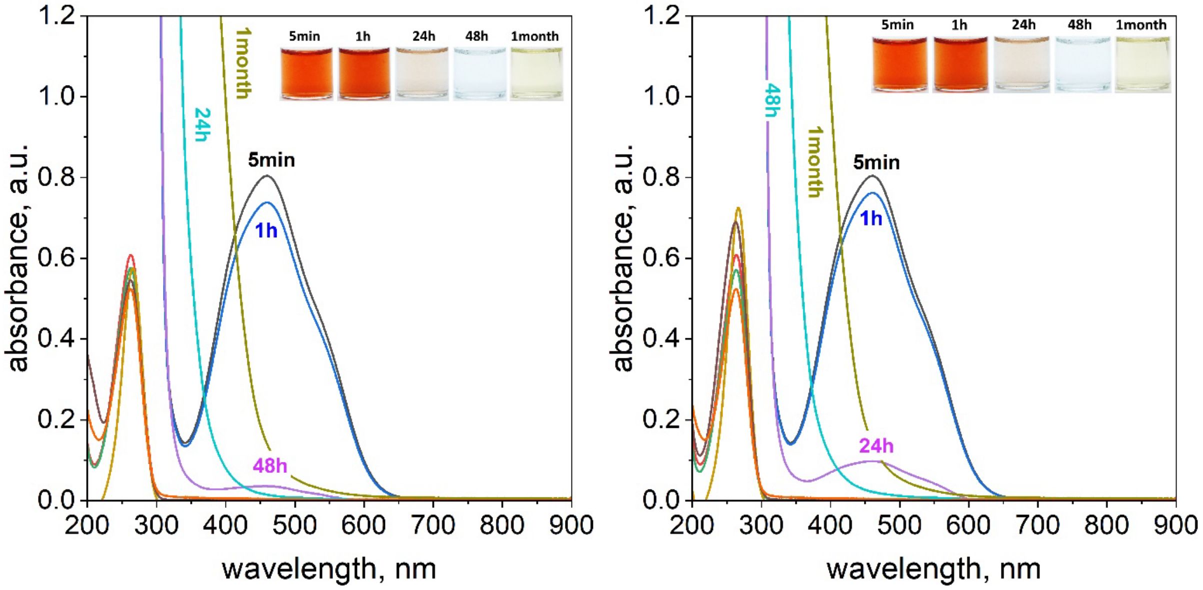

(a) (b)

Figure S15. The UV-Vis spectra of the solution containing the mixture of 4mL of tropaeolin OO (TR) and 0.4 g of ascorbic acid at different exposure to daylight: daylight exposition (DLE) (a); daylight protection (DLP) (b). Conditions: $C_{0,TR} = 5 \cdot 10^{-5}$ mol/dm^3 (the value of concentration before mixing), T = 20°C, path length 1 cm.

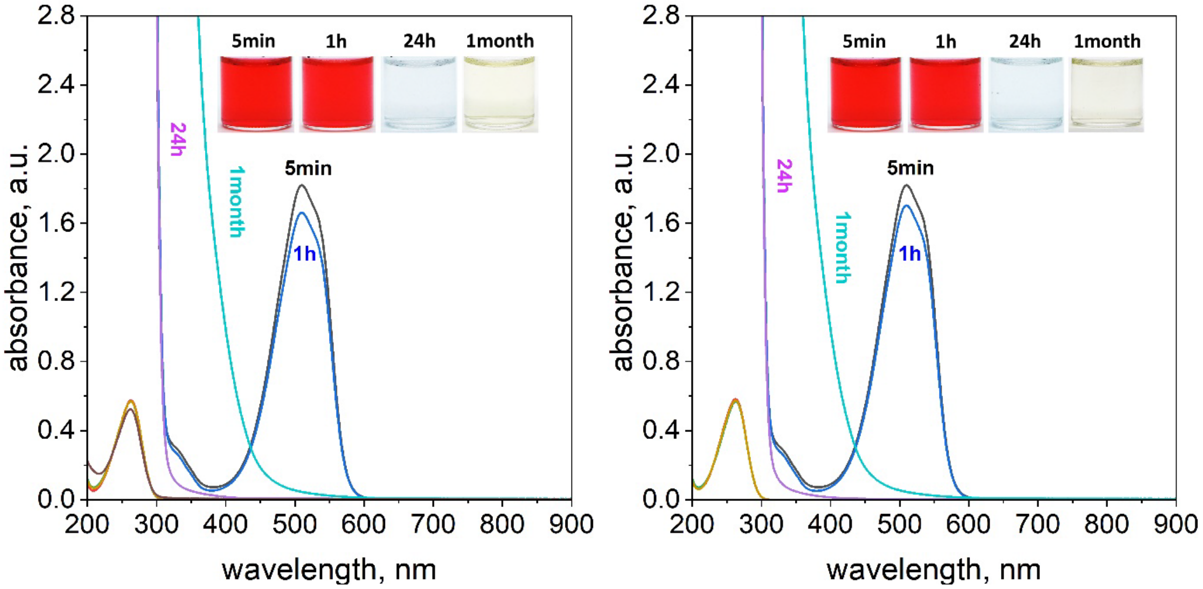

(a) (b)

Figure S16. The UV-Vis spectra of the solution containing the mixture of 4mL of methyl orange (OM) and 0.4 g of ascorbic acid at different exposure to daylight: daylight exposition (DLE) (a); daylight protection (DLP) (b). Conditions: $C_{0,OM} = 5 \cdot 10^{-5}$ mol/dm^3 (the value of concentration before mixing), T = 20°C, path length 1 cm.

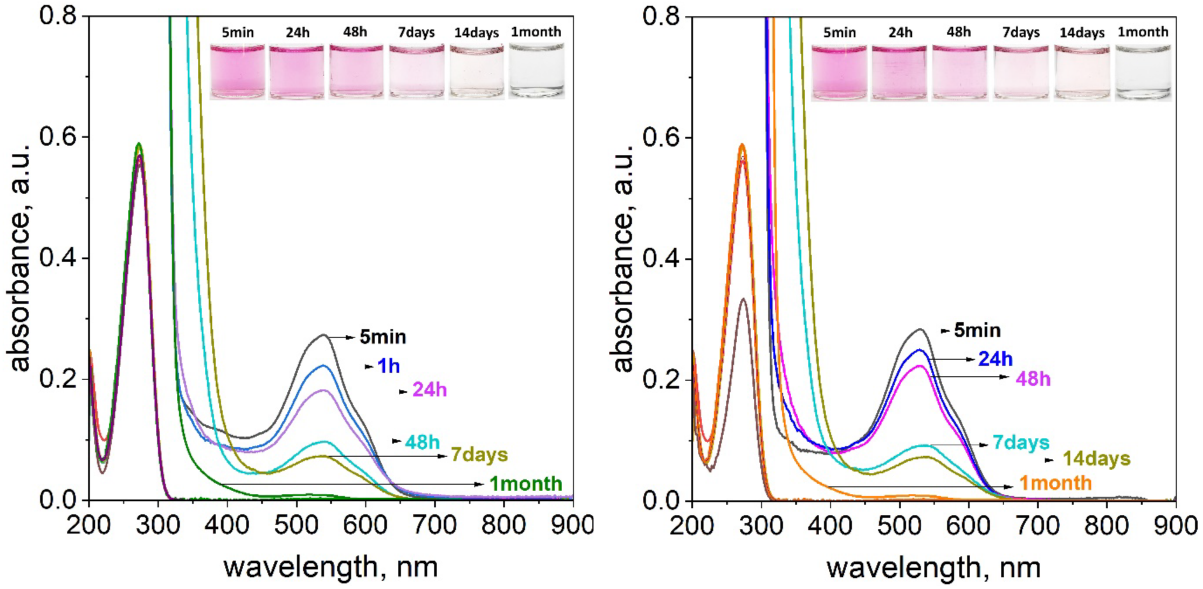

(a) (b)

Figure S17. The UV-Vis spectra of the solution containing the mixture of 4mL of calcon (CL) and 0.4 g of ascorbic acid at different exposure to daylight: daylight exposition (DLE) (a); daylight protection (DLP) (b). Conditions: $C_{0,CL} = 5 \cdot 10^{-5}$ mol/dm^3 (the value of concentration before mixing), T = 20°C, path length 1 cm.

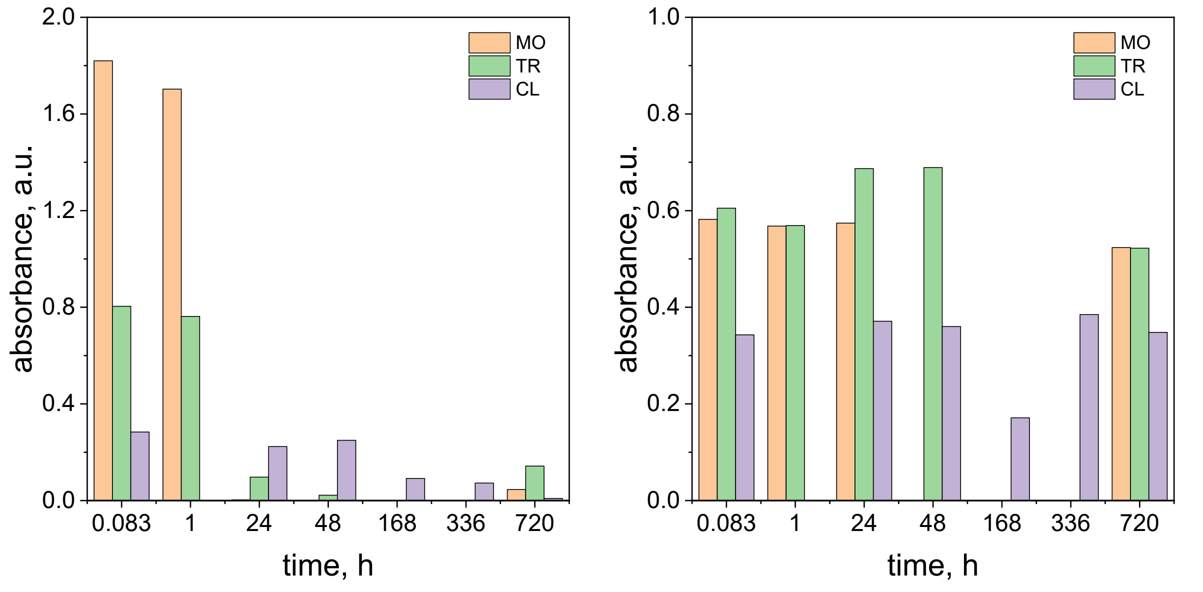

(a) (b)

Figure S18. The change of the absorbance value coming from OM, TR and CL (a) and ascorbic acid (after 10,000 times dilution) (b) with time, at daylight protection (DLP) and constant concentration of ascorbic acid (0,4g). Conditions: $C_{0,MO,TR,CL} = 5 \cdot 10^{-5}$ mol/dm^3 (the value of concentration before mixing), T = 20°C, path length 1 cm.

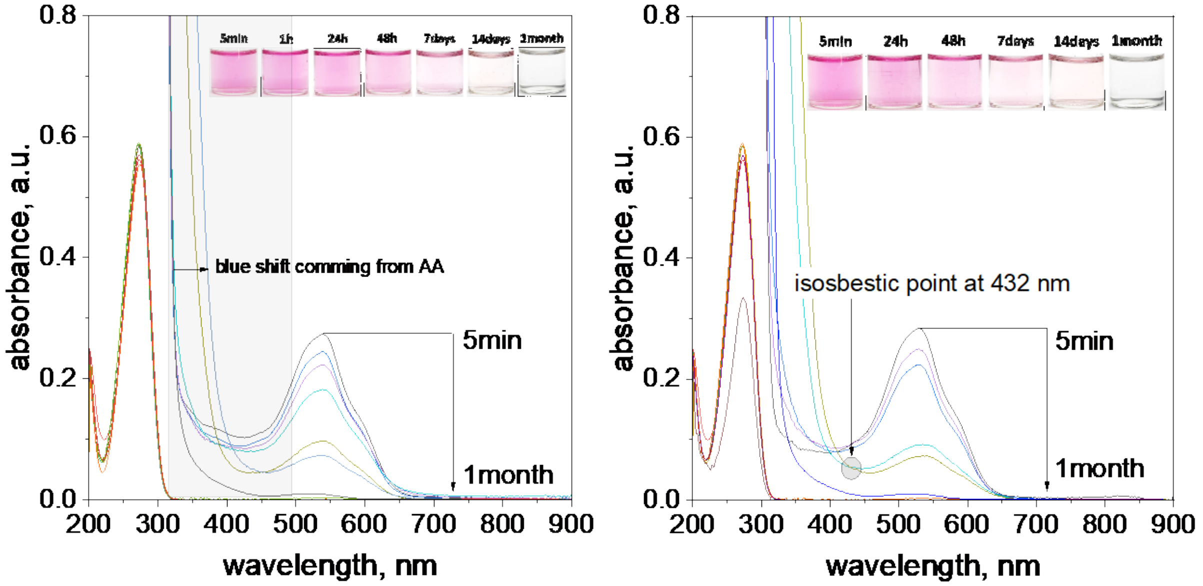

(a) (b)

Figure S19. The UV-Vis spectra of the solution containing the mixture of 4 mL of calcon (CL) and 0.4 g of ascorbic acid at different exposure to daylight: daylight exposition (DLE) (a); daylight protection (DLP) (b). Conditions: $C_{0,CL} = 5 \cdot 10^{-5}$ mol/dm^3 (the value of concentration before mixing), T = 20°C, path length 1 cm.

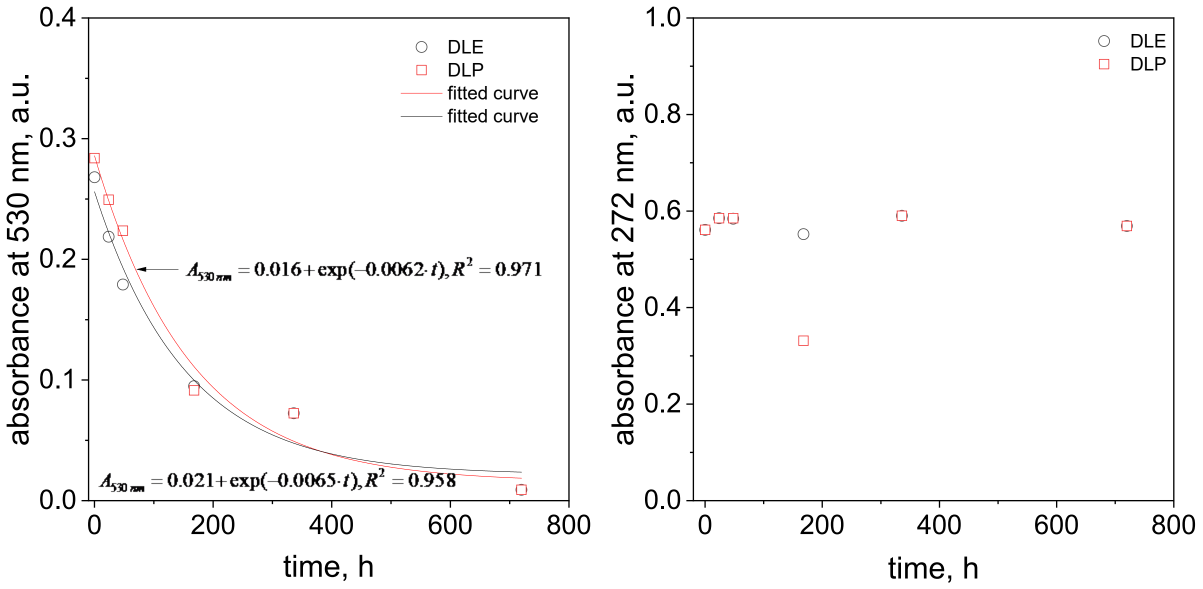

(a) (b)

Figure S20. The kinetic curves registered for calcon at 530 nm (a) with fitting equation to obtained experimental data for DLE and DLP sample; The kinetic curves registered at 272 nm (b) for DLE and DLP sample. Conditions: $m_{ascorbic\ acid}$= 0.4 g (0.57 mol/dm^3), $C_{0,CL} = 5 \cdot 10^{-5}$ mol/dm^3, T = 20°C, path length 1 cm.

S6. The mechanism of azo dyes degradation using ascorbic acid

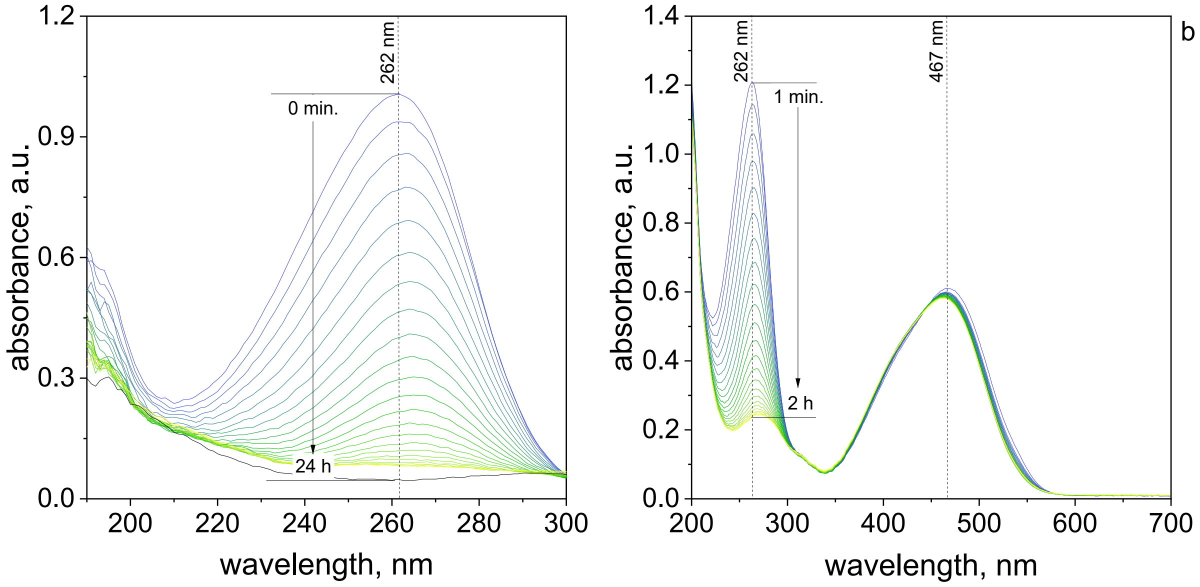

(a) (b)

Figure S21. The UV-Vis spectra evolution (2 h, step 5 min.) registered at 50°C for solutions containing: ascorbic acid (AA-OX) (a); ascorbic acid and MO (AA-MO) (b). Conditions: volumetric ratio: 2mL MO (or water): 2 mL ascorbic acid, $C_{0,\,MO} = 5\cdot 10^{-5}$ mol/dm^3, $C_{0,\,ascorbic\ acid} = 2\cdot 10^{-4}$ mol/dm^3.

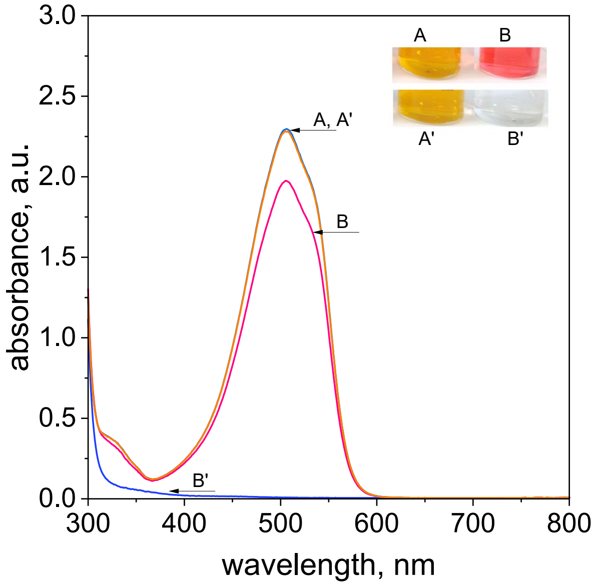

Figure S22. A – sample containing MO and H_2O_2 (t = 2 min.); A' – sample containing MO and H_2O_2 (t = 30 min.); B – sample containing MO, AA and H_2O_2 (t = 2 min.); B' – sample containing MO, AA and H_2O_2 (t = 30 min.). Conditions: $C_{0,\,MO} = 5\cdot 10^{-5}$ mol/dm^3, mAA = 0.025 g, V_{MO} = 4 mL, T = 20°C.

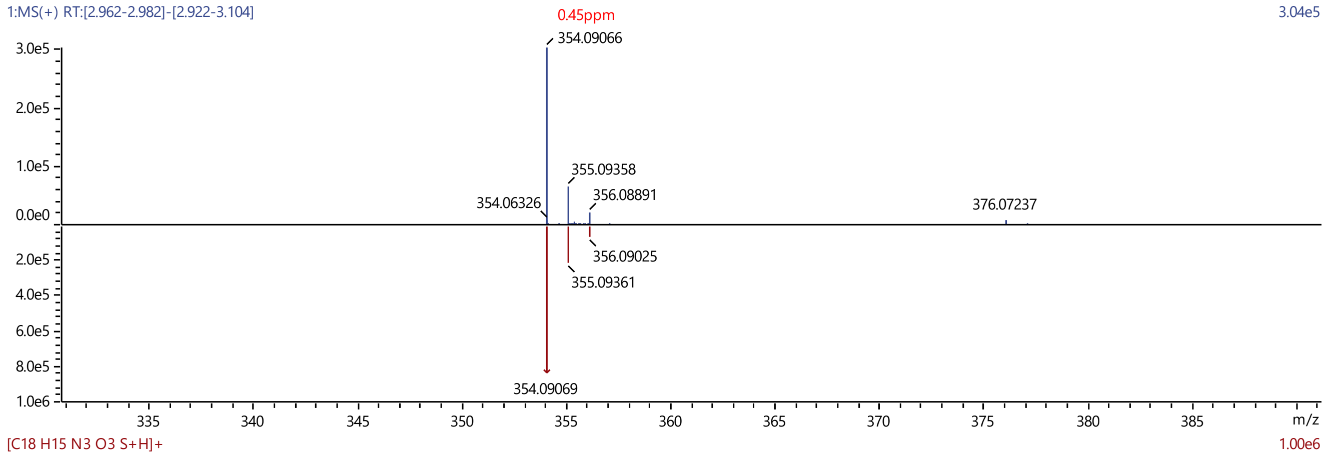

Figure S23. MS spectrum of TR solution containing ascorbic acid after 5 min. Conditions before dilution: $C_{0,\ TR} = 5\cdot 10^{-5}$ mol/dm^3, $m_{ascorbic\ acid} = 0.2$ g , T = 20°C. Sample was diluted before experiment 10,000.

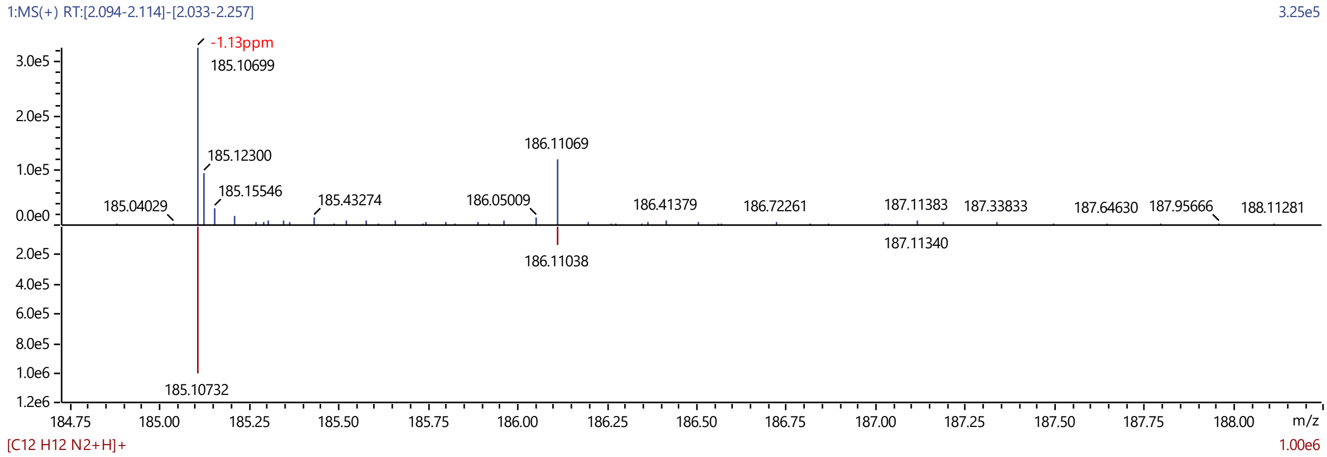

Figure S24. The MS spectrum of the TR degradation products (7 days later).

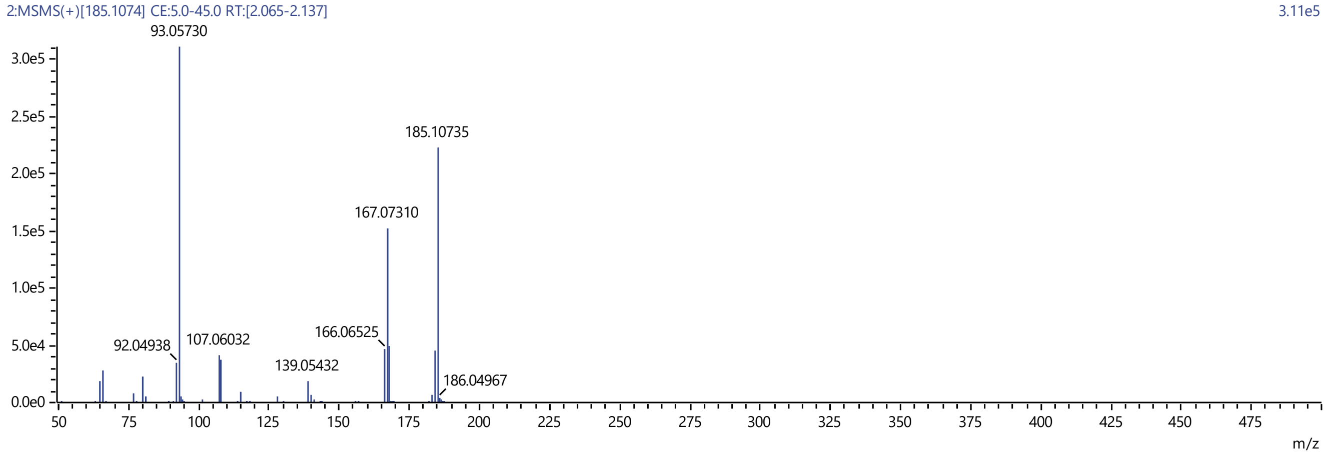

Figure S25. The MS2 spectrum (after fragmentation).
